# Markov Chain Ontology Analysis (MCOA)

**DOI:** 10.1186/1471-2105-13-23

**Published:** 2012-02-03

**Authors:** H Robert Frost, Alexa T McCray

**Affiliations:** 1 Center for Biomedical Informatics, Harvard Medical School, Boston, MA 02115, USA

## Abstract

**Background:**

Biomedical ontologies have become an increasingly critical lens through which researchers analyze the genomic, clinical and bibliographic data that fuels scientific research. Of particular relevance are methods, such as enrichment analysis, that quantify the importance of ontology classes relative to a collection of domain data. Current analytical techniques, however, remain limited in their ability to handle many important types of structural complexity encountered in real biological systems including class overlaps, continuously valued data, inter-instance relationships, non-hierarchical relationships between classes, semantic distance and sparse data.

**Results:**

In this paper, we describe a methodology called Markov Chain Ontology Analysis (MCOA) and illustrate its use through a MCOA-based enrichment analysis application based on a generative model of gene activation. MCOA models the classes in an ontology, the instances from an associated dataset and all directional inter-class, class-to-instance and inter-instance relationships as a single finite ergodic Markov chain. The adjusted transition probability matrix for this Markov chain enables the calculation of eigenvector values that quantify the importance of each ontology class relative to other classes and the associated data set members. On both controlled Gene Ontology (GO) data sets created with Escherichia coli, Drosophila melanogaster and Homo sapiens annotations and real gene expression data extracted from the Gene Expression Omnibus (GEO), the MCOA enrichment analysis approach provides the best performance of comparable state-of-the-art methods.

**Conclusion:**

A methodology based on Markov chain models and network analytic metrics can help detect the relevant signal within large, highly interdependent and noisy data sets and, for applications such as enrichment analysis, has been shown to generate superior performance on both real and simulated data relative to existing state-of-the-art approaches.

## Background

Ontologies have become a crucial component for the analysis, retrieval and integration of the data underpinning modern biomedical science [1]. Whether structured as controlled vocabularies or expressive description logic-based models, biomedical ontologies have been used to manually and semi-automatically annotate enormous volumes of genomic, clinical and bibliographic information. These annotated datasets support a range of ontology-driven applications such as semantic search, enrichment analysis, data integration and clinical decision support.

Of particular importance in the biomedical space are the family of applications, including enrichment analysis [2], semantic similarity clustering [3] and data-based ontology evaluation [4], that quantify the importance of classes in an ontology relative to a collection of domain data. These applications, especially enrichment analysis based on the Gene Ontology (GO) [5], have been widely adopted by the scientific community and have proven effective in distilling large datasets that would otherwise be extremely difficult for researchers to interpret. Yet, despite the extensive use and high utility of these applications, the underlying analytical methods remain limited in their ability to successfully detect and synthesize several important types of ontological and dataset complexity, including class overlaps, continuously valued data, inter-instance relationships, non-hierarchical class relationships, semantic distance and sparse data.

To help address these limitations, we have developed a new methodology, Markov Chain Ontology Analysis (MCOA), for analyzing hierarchical models relative to a collection of domain data. Our approach represents the combination of an ontology and the instances in an associated dataset as a single finite ergodic Markov chain whose adjusted transition probability matrix is used to compute modified eigenvector centralities, or steady-state probabilities, for each class and instance. The negative log of these modified eigenvector centralities, a quantity we call the **information rank **of the class, represents the importance of each class relative to both the data set and other classes in the ontology.

In the remainder of this paper, we outline the analytical challenges that motivated the development of our methodology, detail the mathematical model of our technique and demonstrate its utility in the context of GO enrichment analysis. Following a standard benchmarking process, we demonstrate the ability of a MCOA-based enrichment analysis method to outperform existing state-of-the-art enrichment methods on simulated gene enrichment datasets. To evaluate the performance of MCOA on real experimental data, we compare the enrichment results generated by MCOA with other comparable methods using gene expression data from a study of Parkinson's disease. Finally, we discuss other applications that could benefit from the MCOA approach and our plans for future investigations.

### Enrichment Analysis

Although the analysis approach we propose is relevant to any application that quantifies the importance of ontology classes relative to a dataset, we frame the discussion in this paper in the context of enrichment analysis. Our focus on enrichment analysis is motivated both because of the widespread use of enrichment analysis in the biomedical field as well as the fact that the technical challenges faced by enrichment analysis methods are directly relevant to many other ontology-based data analysis activities.

Enrichment analysis assesses whether classes in an ontology are statistically over or under-represented in a specific dataset based on the semantic annotations of dataset members relative to some baseline distribution. In the biomedical field, enrichment analysis methods are commonly employed to determine the statistical enrichment of GO categories for gene expression data by comparing the annotation frequency in a target gene list with the annotation frequency in a background collection of genes. The widespread use of the method in this context has motivated the extensive manual annotation of genomic and proteomic data with GO categories and the development of a wide range of enrichment analysis techniques and tools [2]. Although analysis relative to GO is the most common use case, the underlying enrichment analysis techniques are relevant to any biomedical ontology (or even flat categorization) and correspondingly annotated dataset (e.g., enrichment of pathways defined in KEGG [6]). Recent successes applying enrichment analysis outside the genomic domain include efforts by Tirrel *et al *[7] who performed enrichment analysis of the ontologies contained in BioPortal [8] relative to both MEDLINE and the collection of biomedical repositories aggregated by the NCBO Resource Index [9].

Whether analyzing genomic data for enrichment of GO categories or bibliographic data for enrichment of classes in a clinical ontology, the same set of enrichment methods can be employed. Huang *et al *[2] decomposed the existing diversity of 68 different enrichment methods into three broad classes: singular enrichment analysis (SEA, Class 1), gene set enrichment analysis (GSEA, Class 2) and modular enrichment analysis (MEA, Class 3). SEA represents the traditional linear enrichment analysis strategy and approaches in this category evaluate ontology classes one-at-a-time for enrichment against a fixed list of interesting dataset members using a statistical test like Fisher's exact test following the hypergeometric distribution. Methods in this class vary according to the statistical test employed, the criteria by which the dataset is selected and any special heuristics or weightings applied during analysis. The GSEA class of methods, which includes the original Gene Set Enrichment Analysis (GSEA) technique [10]) as well as the more recent Random sets [11] and LR Path [12] methods, take advantage of experimentally derived weights to evaluate the entire dataset. Methods in the MEA category evaluate the enrichment of multiple ontology classes simultaneously by taking into account the full network of ontology and dataset relationships. Similar to the methods in the SEA category, most MEA methods do not consider continuous instance weights and must therefore be run against a fixed list of interesting data set members.

The MEA category includes the MCOA-based enrichment analysis approach described in this paper as well as a number of state-of-the-art techniques developed since the publication of the Huang *et al *[2] survey such as NOA by Wang *et al *[13], TopoGSA by Glaab *et al *[14], GenGO by Lu *et al *[15] and MGSA by Bauer *et al *[16,17]. NOA attempts to capture the functional enrichment of inter-instance relationships through the calculation of link annotations and subsequent application of standard statistical enrichment methods to these annotations (e.g., hypergeometric distribution). TopoGSA supports the visualization and analysis of network analytic properties for gene and protein sets mapped to interaction networks. GenGO and MGSA, which both adopt a generative probabilistic model of gene activation, are particularly well suited to the challenge of class overlaps in the presence of noise and are among the best methods in terms of benchmarked enrichment performance. GenGO uses the generative model and a maximum likelihood approach to identify a small set of GO categories that best explains an observed experimental gene list. P-values for this optimal set of GO categories are then computed using the standard Fisher's exact test, or any other desired test statistic, including optional multi-hypothesis correction. Motivated by the GenGO approach, Bauer *et al *[16] also adopted a generative model of gene activation for their MGSA method. MGSA uses a Bayesian network to model gene activation and represents GO enrichment using the marginal posterior probabilities of each GO category computed using a Markov Chain Monte Carlo algorithm. Rather than identifying a fixed set of classes that maximize the objective function based on the generative model, MGSA provides a posterior probability enrichment score for all classes. Although not directly comparable against GSEA methods, when evaluated against existing SEA and MEA methods, the GenGO and MGSA methods are significantly better, as measured on synthetic data, at correctly identifying enriched GO categories while minimizing reported false positives and false negatives [15,16].

Despite the extensive use and high utility of enrichment analysis applications and the important recent advances made in the GSEA and MEA categories, existing analytical methods remain limited in their ability to successfully analyze the full spectrum of ontological and dataset complexity. Challenging structural features include overlaps between ontology classes, continuous instance and annotation weights, relationships between instances, non-hierarchical relationships between classes, semantic distance and sparse data. These analytical challenges, and how current enrichment methods attempt to address them, are discussed in further detail below.

### Analysis Challenges

#### Class overlaps

Methods in the SEA and GSEA categories commonly generate enrichment results comprising long lists of highly correlated classes, leaving users to determine which of multiple, largely redundant, classes are actually relevant. This problem is due to both the overlaps between class members and the fact that SEA and GSEA methods evaluate each class independently for enrichment and thus fail to take class interdependencies into account. Overlaps between the member sets of different classes can result from several structural features:

• **Inheritance**: one class is an ancestor of the other class and therefore all dataset members annotated to the descendant are implicitly annotated to the ancestor.

• **Multiple parents**: both classes share a common descendant and therefore are implicitly annotated with the same dataset members.

• **Multiple annotations**: a dataset member is annotated to both classes (or descendants of both classes).

Overlaps between classes are very common in practice with each GO term overlapping with an overage of 1078 other terms based on common human gene annotations (see Additional File 1 for details). When overlaps between enriched classes exist because of multiple annotations, the results are also skewed in favour of instances associated with a large number of classes. This distortion can be particularly problematic for cases where annotation bias exists (e.g., protein annotation bias [18]) or cases where the total amount of enrichment evidence should be based on the number of instances and their weights rather than on the number of annotations (e.g., web page ranking using the PageRank algorithm [19]).

The class overlap problem has been explored by several existing enrichment analysis approaches including MGSA, GenGO, parent-child union by Grossmann *et al *[20] and elim and weight by Alexa *et al *[21]. The parent-child union, elim and weight methods all address overlaps by computing statistical enrichment using the hypergeometric distribution with counts weighted according to the hierarchical structure of the ontology. Parent-child union computes enrichment for a specific class in the context of dataset members annotated to the parents of the class. Elim removes genes annotated against enriched subclasses when computing enrichment for parent classes and weight generalizes the elim approach by adjusting gene weight to a value between 0 and 1. Because the weighting heuristics used by parent-child union, elim and weight utilize just the structure of the ontology, these methods only address overlaps due to inheritance or multiple parents. Although GenGO and MGSA are able to detect all cases of overlaps, the fact that these methods collapse the ontology hierarchy means that they are unable to distinguish between the different cases of overlap, which impacts support for semantic distance and annotation bias.

#### Continuously valued data

A key drawback of methods in the SEA category and most methods in the MEA category is their inability to model continuously valued data. For most biological data of interest in an enrichment analysis scenario, dataset members have varying levels of experimental significance and continuous weights can be associated directly with each instance (e.g., differential gene expression, test statistic associated with SNP-to-gene analysis, etc.) or with each instance-to-class annotation (e.g., probabilistic confidence score generated via statistical classification, GO annotations weighted according to source of evidence, etc.). Continuous weights can also be associated directly with classes or with inter-instance and inter-class relationships (e.g., protein-protein interaction scores, gene co-expression scores, etc.). Analyzing continuously valued datasets using SEA or MEA methods requires the use of an arbitrary cut-off with all dataset members or annotations above the cut-off given equal weighting in the analysis, potentially leading to significantly skewed enrichment results. Addressing this shortcoming is the primary objective of methods in the GSEA category including Gene Set Enrichment Analysis (GSEA) [10], which computes statistical significance for all genes in all differentially expressed arrays using a weighted Kolmogorov-Smirov test; LRPath [12], which uses a logistic regression likelihood ratio test compute significance of enrichment for all genes taking expression level into account; Random-sets [11], which incorporates quantitative instance scores to compute class enrichment values using an analytical approximation of the statistical distribution and is asymptotically equivalent to the LRPath technique; and ProbCD [22], which supports probabilistic instance and annotation weights and computes statistical significance using Goodman-Kruskal gamma and comparison against a null distribution estimated via random permutations.

Although the GSEA methods avoid a potentially arbitrary dataset "cut-off" through the use of continuous dataset weights, this requirement can be problematic in cases where a single biologically meaningful value for each gene does not exist. GSEA methods are further limited by their one-at-a-time analysis of ontology classes and, in practice, have been found to generate enrichment results very similar to those output by SEA methods on actual experimental data [2].

#### Inter-instance relationships

Meaningful relationships often exist between the members of the datasets targeted for enrichment analysis (e.g., citation links between publications, protein-protein interaction links, gene-gene links in gene regulatory networks, etc.). Network models are particularly well suited for representing the interconnections in real biological systems [23-25]. Similar to the links in a social network or hyperlinks between web pages, such instance-level relationships provide evidence of a relative ranking between instances that can be quantified using network analysis metrics such as eigenvector centrality. The use of such network analysis techniques is commonly performed on biomedical networks comprising data instances. Although the output from this type of analysis can be used to adjust the weight of genes for subsequent enrichment analysis using GSEA category methods capable of handling continuous values, current state-of-the-art methods do not compute or use such metrics for enrichment analysis. While the NOA method of Wang *et al *[13] does directly focus on the relationships between dataset members, the goal of this approach is a functional analysis of the gene-to-gene links themselves rather than the use of gene-to-gene links to adjust the functional enrichment of specific genes. Analysis of datasets lacking links between dataset members is not possible with NOA.

#### Non-hierarchical class relationships

Standard enrichment analysis only considers hierarchical relations between classes (is-a, part-of), however, many relevant biomedical ontologies, including GO, include non-hierarchical class relationships (e.g., regulates). Accounting for such inter-class relationships may be even more relevant in scenarios where multiple inter-related ontologies are jointly analyzed and inter-class relationships are used to capture mappings between classes in different ontologies (e.g., relationship between GO categories and KEGG pathways). Although the same network analytical methods used to analyze instance-level links can be applied on the ontology graph, the current set of state-of-the-art enrichment methods do not do so, and, for most enrichment approaches, their incorporation is not feasible due to the nature of the underlying statistical tests.

#### Semantic distance

When analyzing data against hierarchical ontologies, it is generally desirable to bias more specific classes over more general classes when both classes are associated with the same number of dataset members. Standard SEA category methods like Fisher's exact test measure significance based solely on annotation frequency and ignore semantic distance. Although semantic distance is incorporated into methods such as parent-child union, elim and weight, the state-of-the-art MEA methods GenGO and MGSA use flattened representations of the ontology and therefore fail to explicitly incorporate semantic distance.

#### Sparse data

Real datasets frequently suffer from sparsity due to a variety of data collection and experimental design issues [26]. Bayesian approaches, which incorporate prior probabilities based on knowledge about the likely statistical distribution of the data, are better able to handle sparse data then frequentist approaches like those based on Fisher's exact test, which need to employ some type of smoothing (e.g., Laplace or add one smoothing). Bayesian methods that perform enrichment analysis using a prior probability distribution include MGSA and the BayGO framework [26]. Although these Bayesian methods enable the enrichment analysis of sparse data, their lack of support for inter-instance relationships, non-hierarchical class relationships and semantic distance means that only a limited range of sparse datasets can currently be analyzed.

## Methods

Our approach represents the combination of the classes in an ontology and the instances in an associated dataset as a single finite ergodic Markov chain whose adjusted transition probability matrix is used to compute modified eigenvector centralities, or steady-state probabilities, for each class. These modified eigenvector centralities, a quantity we term the information rank, provide a measure of the importance of each class relative to both a dataset and the other classes in the ontology. Similar to annotation frequency, the information rank of a class can be used to support applications that compare the importance of a class in a target dataset with a baseline dataset (e.g., enrichment analysis).

### Ontology Model

For defining our approach and discussing other related methods, we follow Bade *et al *[27] and Cimiano *et al *[28] and adopt a simplified formal model of an ontology and its extension as a rooted hierarchy with instance assignments. Although both our analysis approach and many related techniques can be generalized to more complex structures, as formalized by the description logic-based models [29] used for popular ontology modelling languages such as OWL, this minimal structure contains the essential modelling primitives for evaluating GO enrichment analysis and allows the methodology to be developed with minimal descriptive complexity. Definitions 1 and 2 below formally define the ontology model. Potential extensions to this model include class weights, weights for inter-class relationships, weights for instance-to-class relationships and weights for inter-instance relationships.

***Definition 1 (Ontology)***: *An ontology is a directed acyclic graph of classes structured in a hierarchy and represented by the tuple O *= 〈*C, parent*(*c*)〉

• ***C ****is a non-empty set class identifiers*

• *A strict partial ordering relation **parent(c) **that maps each class c in C to the set of direct parents of c in the class hierarchy*. ∀*c *∈ *C: parent*(*c*) ⊆ *C*

***Definition 2 (Ontology Extension)***: *The extension of an ontology is represented by the tuple E *= 〈*I, type*(*i*), *rel*(*i*), *weight*(*i*)〉

• *A potentially empty set **I **of instance identifiers*

• *An instance type relation **type(i) **that maps each instance in I to a set of one or more classes in C*. ∀*i *∈ *I: type*(*i*) ⊆ *C*

• *An inter-instance relation **rel(i) **that maps each instance in I to a set of zero or more other related instances in I*. ∀*i *∈ *I: rel*(*i*) ⊆ *I*

• *An instance weight relation **weight(i) **that maps each instance in I to a normalized weight between 0 and 1*. ∀*i *∈ *I: weight*(*i*) ∈ 0[1]

### Markov Chain Model

Our proposed methodology for analyzing an ontology relative to a collection of domain data represents the combination of an ontology and its extension as a finite ergodic Markov chain. A finite Markov chain is a finite stochastic process in which the probability of transitioning from a state i to a state j is only dependent on the state i and not on the path taken through the chain to arrive at state i [30]. This property of a Markov chain is called the Markov property and, for an ergodic Markov chain, it enables the state transitions to be represented as a stochastic matrix with the special property of possessing a principal left eigenvector for the maximum eigenvalue of 1. The components of this principal left eigenvector represent the steady-state probabilities for each state in the chain. Definition 3 below provides the formal specification of a Markov chain.

***Definition 3 (Finite Ergodic Markov Chain)***: *A finite ergodic Markov chain is a finite stochastic process characterized by:*

• *A non-empty set of states S of size N*

• *An N × N transition probability matrix P where each entry p_ij _represents the probability that the state will be j if the current state is i*.

• *By the Markov property, the transition probability values p_ij _are only dependent on the current state i. Therefore:*

$$\forall i,j,P_{ij}\in \left[0,1\right]$$

$$\forall i,\overset{N}{\underset{j=1}{\sum }}P_{ij}=1$$

• *The transition probability matrix for a Markov chain is a stochastic matrix with a principal left eigenvector, $\vec{e}$, of length N for its largest eigenvalue of 1*.

• *For a finite ergodic Markov chain, the components of this principal left eigenvector are the steady-state probabilities, or eigenvector centralities, of the states of the Markov chain*.

### Core MCOA Process

At the core of our methodology is a process for computing an eigenvector-based score for each class in an ontology relative to an extension of that ontology (i.e., a collection of data annotated using the ontology classes). We call this the information rank based on its similarity to the well-known PageRank algorithm for computing the ranks of web pages using a Markov model of a random walk with jumps through web page links [19]. The MCOA process involves three key steps:

• **Step 1**: Model the ontology and extension as a single finite ergodic Markov chain.

• **Step 2**: Create an adjusted transition probability matrix for the Markov chain.

• **Step 3**: Use the transition probability matrix to compute the eigenvector-based steady-state probability and information rank for each ontology class.

**Figure 1:**
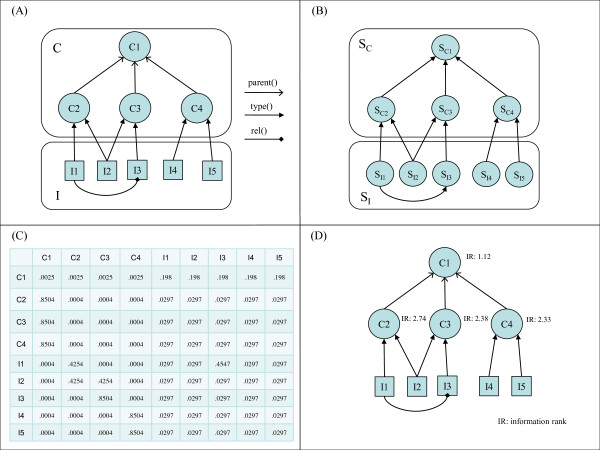
**MCOA mapping between ontology, ontology extension and Markov chain**. (A) Simple ontology and extension. (B) Markov chain representing simple ontology and extension according to MCOA method (C) Adjusted transition probability matrix for Markov chain according to MCOA method (D) Information rank values generated from adjusted transition probability matrix using α = 0.15 and ω = 0.01.

Algorithmic details for each of these steps are outlined below and formalized in Definitions 4, 5, 6 and 7. Figure 1 illustrates these steps for a simple ontology.

#### Step 1: Model Ontology and Extension as Markov Chain

Our approach builds a Markov chain model of an ontology and its extension by mapping classes in the ontology and the instances of those classes to states in the Markov chain and by mapping all instance-to-class relations and hierarchical relations between classes to state transitions. Given the simplified model of an ontology and its extension specified in Definitions 1 and 2 and the model of a finite ergodic Markov chain specified in Definition 3, the process for building a Markov chain from an ontology and its extension is formalized in Definition 4 below. Figure 1B shows an example Markov chain for the ontology in Figure 1A generated according to this mapping.

***Definition 4 (Ontology-to-Markov Chain Mapping)***: *The mapping between an ontology O and its extension E (as defined in Definitions 1 & 2) and a finite ergodic Markov chain is characterized by:*

• *A partitioning of the set of Markov chain states into two disjoint subsets S_C_, which contains the states corresponding to ontology classes, and S_I_, which contains the states corresponding to ontology instances:*

$$S=S_{C}\cup S_{I},S_{C}⊄S_{I}$$

• *Equivalence mapping class(s) (and inverse mapping state(c)) between the states in subset S_C _of the Markov chain and the classes in set C (i.e., there is a one-to-one mapping between each class and each Markov chain state in S_C_)*.

• *Equivalence mapping inst(s) between the states in subset S_I _of the Markov chain and the instances in set I (i.e., there is a one-to-one mapping between each instance and each Markov chain state in S_I_)*.

#### Step 2: Create Adjusted Transition Probability Matrix

Calculating the transition probability matrix for the Markov chain defined above involves three key adjustments:

• A random jump probability α. This is equivalent to the damping factor, d, used in the PageRank algorithm, specifically α = 1-d.

• A parameter, ω, that controls how much of the random jump probability is distributed among class states, S_C_, vs. instance states, S_I_

• The weights of each individual instance, as specified by the function weight(i)

Using these parameters, the creation of the adjusted transition probability matrix can be formalized according to Definition 5 below. Figure 1C contains the adjusted transition probability matrix created for the ontology in Figure 1A according to this process.

***Definition 5 (Adjusted Transition Probability Matrix)***: *The adjusted transition probability matrix P for the finite ergodic Markov chain that represents an ontology and its extension, as specified in Definition 4 above, is defined by:*

• *A random jump parameter*, α*, which determines the probability that the Markov chain makes a random jump to one of the other states rather than following the defined transitions from that state*.

• *A probability distribution weight*, ω*, that determines how probabilities are distributed between states representing classes, S_C_, and states representing instances, S_I_, following each random jump. If *ω *= 0, random jump probability is distributed only among instance states, likewise, if *ω *= 1, random jump probability is distributed only among class states*.

• *The instance weight function weight(i), which is used to compute a potentially non-uniform distribution of random jump probabilities among the instances*.

• *Given the definitions above, the entries p_ij _of the N × N transition probability matrix P are defined as follows (where i represents the source state and j represents the destination state of the transition):*

$$P_{ij}=\left\{\begin{array}{c} s_{i}\in S_{C}:\left\{\begin{array}{c} s_{j}\in S_{C}:\left\{\begin{array}{c} class\left(s_{j}\right)\in parent\left(class\left(s_{i}\right)\right):\frac{1-\alpha }{|parent\left(class\left(s_{i}\right)\right)|}+\frac{\alpha \omega }{|S_{C}|}\\ class\left(s_{j}\right)\notin parent\left(class\left(s_{i}\right)\right):\frac{\alpha \omega }{|S_{C}|} \end{array}\right\}\\ s_{j}\in S_{I}:\frac{\alpha \left(1-\omega \right)weight\left(inst\left(s_{j}\right)\right)}{\underset{n\in I}{\sum }weight\left(n\right)} \end{array}\right\}\\ s_{i}\in S_{I}:\left\{\begin{array}{c} s_{j}\in S_{C}:\left\{\begin{array}{c} class\left(s_{j}\right)\in type\left(inst\left(s_{i}\right)\right):\frac{1-\alpha }{|type\left(inst\left(s_{i}\right)\right)+rel\left(inst\left(s_{i}\right)\right)|}+\frac{\alpha \omega }{|S_{C}|}\\ class\left(s_{j}\right)\notin type\left(inst\left(s_{i}\right)\right):+\frac{\alpha \omega }{|S_{C}|} \end{array}\right\}\\ s_{j}\in S_{I}:\left\{\begin{array}{c} inst\left(s_{j}\right)\in rel\left(inst\left(s_{i}\right)\right):\frac{1-\alpha }{|type\left(inst\left(s_{i}\right)\right)+rel\left(inst\left(s_{i}\right)\right)|}+\frac{\alpha \left(1-\omega \right)weight\left(inst\left(s_{j}\right)\right)}{\underset{n\in I}{\sum }weight\left(n\right)}\\ inst\left(s_{j}\right)\notin rel\left(inst\left(s_{i}\right)\right):\frac{\alpha \left(1-\omega \right)weight\left(inst\left(s_{j}\right)\right)}{\underset{n\in I}{\sum }weight\left(n\right)} \end{array}\right\} \end{array}\right\} \end{array}\right\}$$

The use of the random jump and non-uniform distribution parameters defined above has several benefits in the context of our method:

• It ensures that the Markov chain is ergodic (it would otherwise be absorbing given the 0 out-degree for any root node).

• It allows for prior probability smoothing. Classes without instances can be assigned a configurable portion of the random jump probability as a form of prior probability smoothing. By varying the ω parameter between 0 and 1, the relative weight of a uniform prior probability distribution can be adjusted relative to the analyzed dataset distribution.

• It enables the use of class and instance weighting. Similar to the topic-sensitive PageRank approach [31], a non-uniform distribution of random jump probabilities can be used to mirror differential class and instance weights.

• It allows semantic distance to be quantified. The amount of transferred rank naturally decays as one moves up the hierarchy.

#### Step 3: Compute Information Rank

Given an adjusted transition probability matrix as specified in Definition 5 above, the importance of each class relative to the dataset can be quantified using the components of the principal left eigenvector that correspond to classes in the ontology. These eigenvector components represent the steady-state probabilities of the class states in the associated Markov chain. Normalizing these steady-state probabilities relative to the probabilities for all class states and then taking the negative log of the normalized probabilities generates the information rank. The definitions of steady-state class probability and information rank are formalized in Definitions 6 and 7 below. Figure 1D shows the information rank values for the example ontology in Figure 1A.

***Definition 6 (Adjusted Steady-State Class Probability)***: *Given the definitions above, the adjusted steady state probability for a class c in C is defined as the ratio of the principal left eigenvector component for the Markov chain state corresponding to that class divided by the sum of all class eigenvector components:*

$$\forall c\in C:ssp\left(c\right)=\frac{{\vec{e}}_{state\left(c\right)}}{\underset{s\in S_{C}}{\sum }{\vec{e}}_{s}}$$

***Definition 7 (Information Rank)***: *The information rank for a class c in C is defined as the negative base-2 log of the adjusted steady-state probability:*

$$\forall c\in C:ir\left(c\right)=-{\text{log}}_{2}\left(ssp\left(c\right)\right)$$

### MCOA Enrichment Analysis

Our initial application of the MCOA method to enrichment analysis adopts the probabilistic generative model of gene activation used by both GenGO and MGSA. It specifically extends the GenGO maximum likelihood approach by adding MCOA-based terms to the objective function used in the original GenGO algorithm. Although our initial enrichment analysis method extends GenGO, MCOA can be integrated with other enrichment methods or used directly to determine enrichment significance by employing permutation tests to compute a distribution of possible information rank values. Our choice of GenGO as a base approach was motivated by several factors:

• **GenGO is one of the best state-of-the-art methods**. GenGO and MGSA are two state-of-the-art MEA approaches shown to provide overwhelmingly superior enrichment performance on simulated data.

• **GenGO is feasible to extend**. Integration of MCOA through modification of the objective function was both feasible and straightforward.

• **GenGO returns intuitive results with flexible statistics**. The GenGO process outputs p-values, using the statistical test of choice, for the set of categories that maximize the log likelihood objective function. Use of p-values, as opposed to the marginal posterior probabilities used by MGSA, make the results of this method more intuitive to researchers and more easily comparable to the results from other enrichment methods. Use of multiple hypothesis correction is also optional.

Execution of the MCOA enrichment analysis algorithm involves three steps:

• **Step 1: **Compute steady state probability scores for the ontology relative to both the reference and target datasets.

• **Step 2**: Find the set of ontology classes that maximizes the likelihood of the observed dataset given a probabilistic generative model.

• **Step 3: **Compute p-values and apply multi-hypothesis correction.

Algorithmic details for each of these steps are outlined below.

#### Step 1: Compute steady state probability scores for the ontology relative to both the reference and target datasets

This step follows the core MCOA process outlined above.

#### Step 2: Find the set of ontology classes that maximize the likelihood of the observed dataset given a probabilistic generative model

The MCOA approach modifies the GenGO objective function by replacing the *α*|*C*| term that penalizes the sizes of active GO categories by a term computed from the MCOA-based steady state probability scores for each active category. This modification of the GenGO objective function to incorporate MCOA steady state probability scores as a regularization parameter is similar to approaches taken for SNP selection during GWAS analysis in which the objective function for a stochastic wrapper algorithm is modified to include preprocessed attribute quality estimates [32]. This replacement term, which is equivalent to a weighted log-odds value, still penalizes large sets of active GO categories while also giving a preference to those categories whose steady state probability is larger in the target dataset than in the reference dataset. Where the steady-state probability ratios are equal for two categories, the weighting acts to prefer the category with a greater steady state probability in the target dataset. Similar to the original GenGO method, MCOA optimizes the objective function via a greedy search algorithm. Optimization of the p and q values also follows the GenGO approach. Although originally specified in terms of GO categories and genes, this approach can be easily generalized to the generic ontology model outlined earlier in the paper and this generalized description is used in the formal definition of the modified objective function in Definition 8 below.

***Definition 8 (MCOA Objective Function)***: *The MCOA method modifies the GenGO log-likelihood function by replacing the *α|*C*| *regularization term with *$\beta \underset{c\in C}{\sum }\text{log}\left(\frac{ssp{{\left(c\right)}_{tar}}^{2}}{ssp{\left(c\right)}_{ref}}\right)$. *The complete modified objective function is:*

$$L\left(C|p,q,G\right)=|A_{g}|\text{log}p+|A_{n}|\text{log}q+|S_{g}|\text{log}\left(1-p\right)+|S_{n}|\text{log}\left(1-q\right)+\beta \underset{c\in C}{\sum }\text{log}\left(\frac{ssp{{\left(x\right)}_{tar}}^{2}}{ssp{\left(c\right)}_{ref}}\right)$$

where:

• *C is the set of active ontology classes*

• *G is the set of active instances*

• *q is the false positive rate or the percentage of instances not associated with an active ontology class that are activated*

• *(1-p) is the false negative rate or the percentage of instances associated with an active classes that are deactivated*

• *A_g _is the set of active instances annotated with at least one active class*

• *A_n _is the set of active instances not annotated with any active classes*

• *S_g _is the set of annotations (materialized according to the ontology hierarchy) between inactive instances and active classes*

• *S_n _is the set of annotations (materialized according to the ontology hierarchy) between inactive instances and inactive classes*

• *ssp(c)_ref _is the steady state probability for ontology class c computed using the reference dataset*

• *ssp(c)_tar _is the steady state probability for ontology class c computed using the target dataset*.

• *β is a parameter that weights the steady state probability regularization term*.

#### Step 3: Compute p-values and apply multi-hypothesis correction

For the set of ontology classes that maximizes the objective function, p-values can be computed using any desired statistical test. Similar to the original GenGO method, the current implementation of MCOA computes p-values using the hypergeometric distribution. If desired, multiple hypothesis correction methods can also be applied to the generated p-values. An important benefit of this approach is that multiple hypothesis correction only needs to consider the subset of classes that maximize the objective function rather than all classes in the ontology.

### GO Enrichment Analysis of Simulated Data

To demonstrate the utility of the MCOA methodology for enrichment analysis of biomedical data, we compared the performance of the MCOA method against GenGO (the Ontologizer implementation), MGSA, Alexa *et al's *weight method [21], Grossmann *et al's *parent-child union and the standard hypergeometric test for Gene Ontology enrichment of simulated Drosophila melanogaster, Homo sapiens and Escherichia coli data sets. For the GenGO, MGSA, weight, parent-child union and hypergeometric methods, we used the implementations and configurations from the Ontologizer framework that were employed to generate the benchmarking results in Bauer *et al *[16].

**Figure 2:**
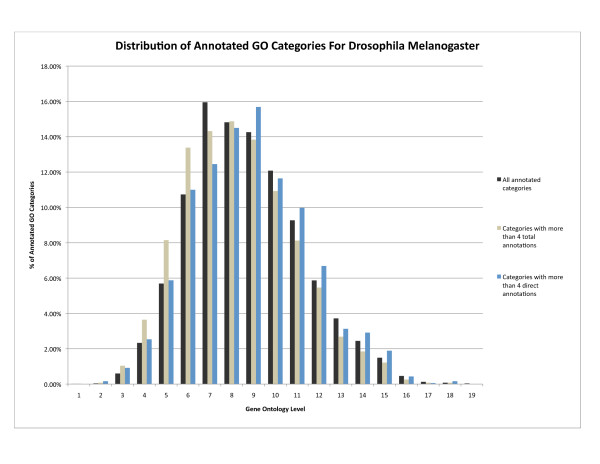
**Distribution of annotated GO categories by hierarchical level**. Distribution of Gene Ontology categories annotated with Drosophila melanogaster genes by hierarchical level. Shown are distributions for all annotated categories, categories with at least 5 total annotations and categories with at least 5 direct annotations.

To enable comparison with prior work, our benchmarking process follows the general approach adopted by Bauer *et al *[16], Lu *et al *[15], Grossmann *et al *[20] and Alexa *et al *[21]. This process builds a test gene list using a pre-selected set of active GO categories, with specific false negative and false positive rates, and then evaluates each enrichment analysis method, using precision/recall metrics, based on its ability to identify the originally selected categories within the noisy dataset. The following parameters control the creation and analysis of the simulated datasets following this approach:

• **Source of GO annotations**: Creation and analysis of the simulated datasets was performed using the following ontology and species annotation files downloaded from the source control repository links on the Gene Ontology website [33]: Gene Ontology (revision 1.2078, 34,171 total GO categories), Drosophila melanogaster annotations from FlyBase [34] (file revision 1.209; 12,966 gene products with 78,094 annotations to GO categories), the Homo sapiens annotations from Go Annotations @ EBI [35] (file revision 1.197; 18,307 gene products with 237,437 annotations to GO categories) and the Escherichia coli annotations from EcoCyc [36] & EcoliHub [37] (file revision 1.57; 3884 gene products with 39,129 annotations to GO categories).

• **Selection of active GO categories: **Following prior work [15,16,20,21] we varied the number of active GO categories between 1 and 5 and avoided selecting hierarchically related categories. Also following prior work [15], we filtered the set of potential active categories to remove categories with fewer than 5 annotations. Such a minimum annotation threshold helps ensure that the selected categories are more likely to be biologically meaningful in the context of experimental data analysis (similar filters are supported on enrichment analysis tools for this same purpose). Whereas Lu *et al *[15] used categories with 5 or more direct or indirect annotations, we have chosen to filter based on just direct annotations. Our motivation for using direct as opposed to total annotations is several-fold:

1. **Generate datasets using a more accurate distribution of categories**. Filtering on the total number of annotations results in the disproportionate removal of leaf categories. For the versions of GO and the Drosophila melanogaster annotations used for our benchmarking, 42.4% of the 7,855 directly and indirectly annotated GO categories are leaf terms. If all categories with fewer than 5 total annotations are removed from this set, the total proportion of leaf categories falls to 20.6% of the remaining 3,953 annotated categories. If filtering is instead based on direct annotations, the proportion of leaf categories remains essentially constant at 43.9% with 1,855 categories left in the set. Both types of filtering effectively maintain the overall distribution of categories by level (see Figure 2) with a correlation coefficient of .986 between the unfiltered distribution and the direct annotation filtering and .981 for total annotation filtering. This pattern is similar for the other evaluated species.

2. **Create simulated datasets that are more consistent with a generative model of gene activation**. Categories with very few or no direct annotations are more likely to be high-level grouping constructs with low analytical value than categories with at least a few direct annotations. A direct annotation for a high-level category provides evidence that the category, rather than one of its subcategories, has been found by curators to provide the best explanation for a specific piece of experimental data. We believe that requiring such evidence for active categories results in datasets that better reflect a generative model of gene activation and represent more biologically meaningful categories.

3. **Create simulated datasets that highlight key analytical challenges**. Filtering based on either direct or total annotations creates a dataset with a high mean annotation level and increased level of class overlaps. Filtering by direct annotations has the added benefit of generating datasets with a larger ratio of direct-to-indirect annotations, highlighting the challenge of differentiating between these types of annotations during enrichment analysis, a distinction ignored by most enrichment methods. With no filtering, each GO category with Drosophila annotations has an average of 7 direct and 61 total annotations. Requiring a minimum of 5 direct annotations results in a set of potentially active categories with an average of 29 direct and 115 total annotations. If a minimum of 5 total annotations is required, the set of active categories has an average of 14 direct annotations and 120 total annotations.

• **False positive rate (q)**: Probability that a gene not associated with an active category is activated. GenGO tested with fairly low false positive rates of 0.01 and 0.15. MGSA reported results for false positive rates of 0.1 and 0.4. The results shown below use a value of 0.1, which corresponds to one of the MGSA values and is between the two GenGO values. Simulations were also performed for false positive rates of .01 and .4 and results can be found in Additional Files 2, 3, 4, 5, 6 and 7.

• **False negative rate (1-p)**: Probability that a gene associated with an active category is deactivated. GenGO reported primary results for false negative rates of 0.1 and 0.5. MGSA reported results for false negative rates of 0.25 and 0.4. The results shown below use a value of 0.25, which matches one of the GenGO settings and is in the between the two MGSA values. Simulations were also performed for false negative rates of .1 and .4 and results can be found in Additional Files 8, 9, 10, 11, 12 and 13.

• **Enrichment threshold for precision/recall calculations (σ)**: The prior benchmarking work by Bauer *et al *[16], Lu *et al *[15] and others computed precision/recall statistics on the rank ordering of analyzed categories irrespective of the actual enrichment significance assigned by the analysis method. Although this is a straightforward evaluation approach that makes comparative evaluation easier, it fails to accurately reflect the performance or actual usage patterns of the underlying enrichment analysis methods. Even though a given method may return all active categories (i.e., 100% recall) with only a few false positives (i.e., high precision), if few of the active categories had enrichment p-values that were significant, a user would have ignored most of these valid results, making the reported precision/recall values misleading. Similar issues also occur when generation of significant enrichment values for the top set of valid categories also results in significant enrichment values for a much larger set of invalid categories. Users analyzing such a result set would need to consider a much larger set of significantly enriched categories despite the high reported precision/recall. Given these factors, we also compared enrichment methods using precision/recall numbers generated using only categories with significant enrichment scores after multiple hypothesis correction. We used a threshold of 0.5 for the MGSA marginal posterior probability, which is the level at which categories are more likely than unlikely according to MGSA (this is the default threshold used for this method in the Ontologizer tool and was the threshold used for MGSA by Bauer *et al *[16] for their analysis of experimental data). For all other methods, we used a p-value threshold of 0.01 after multiple-hypothesis correction using the Bonferroni method.

### GO Enrichment Analysis of Parkinson's Gene Expression Data

To demonstrate the utility of the MCOA method on real experimental data, we compared the enrichment results generated by MCOA, GenGO, MGSA and the standard hypergeometric test on differentially expressed genes from a study of Parkinson's post-mortem brain samples available in the Gene Expression Omnibus (GEO) [38] as dataset GDS3129 [39].

The R GEOquery package [40] was used to retrieve both the raw microarray data and the genes associated with the array platform, which were used as the reference gene list for subsequent enrichment analysis. The set of genes significantly differentially expressed between cases and controls was computed using the R limma [41] package by fitting a linear model, applying empirical bayes shrinkage to compute moderated t-statistics and finally using Benjimani-Hockberg multiple hypothesis correction. Those genes with a false discovery rate below .05 were kept for further analysis and, following the recommendation of Falcon and Gentleman [42], this set was divided based on t-statistic sign into a group whose expression was positively correlated with Parkinson's cases and a group whose expression is negatively correlated with Parkinson's cases. Only the positively correlated group was considered for further analysis with the modified t-statistic values used as a gene weight for the MCOA method. Using the modified t-statistic as a weight enabled us to leverage MCOA's ability to support continuously valued data. Although the MCOA, GenGO and MGSA methods are all able to estimate the false positive rate (q) and false negative rate (1-p) from the data, for this comparison, we ran all methods with fixed false positive and false negative rates of 0.05. For MCOA, the regularization constant β was set to 0.6.

### Implementation

To validate our approach, generate experimental results for this paper and analyze real biomedical data, we have created a prototype implementation of the MCOA core methodology and MCOA enrichment analysis method described above. The core MCOA method was implemented in Java™(version 1.6) using JUNG [43] for the creation of the graphical model and calculation of eigenvector components, Apache Commons Math [44] for basic statistical computations and Jena [45] for processing and reasoning over OWL ontologies [46].

The MCOA-based enrichment analysis method was implemented in Java™ as an extension to the Ontologizer 2 framework [47] and the Ontologizer implementation of the GenGO algorithm. We used the Ontologizer GenGO implementation both to enable comparison with the MGSA benchmarking results and because the original GenGO implementation is not accessible for extension. The benchmarking results reported below were computed using a modification of the Ontologizer benchmarking framework used by Bauer *et al *[16] for evaluating MGSA with additional data processing and statistical computation performed via R.

The MCOA enrichment analysis application can be accessed at the project homepage [48].

## Results

### Analysis Challenge Examples

**Figure 3:**
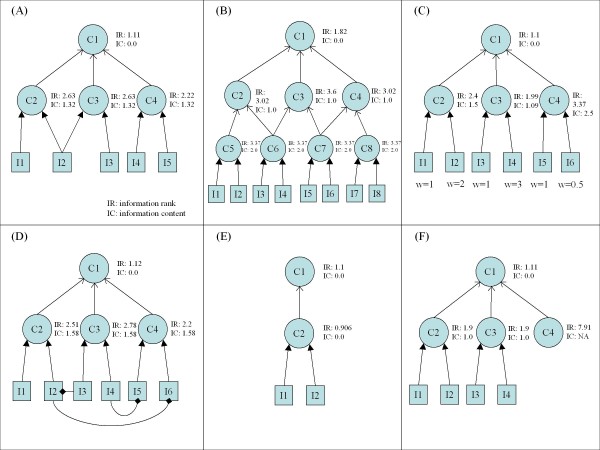
**Analysis challenge examples**. (A) Overlapping classes due to multiple annotations. (B) Overlapping classes due to multiple parents. (C) Continuously valued instance weights. (D) Inter-instance relationships. (E) Semantic distance. (F) Sparse data. For all examples, MCOA run with α = 0.15 and ω = 0.01.

To illustrate the computational behaviour of the MCOA method and the ability of this method to detect complex structural features, we computed information rank and information content values for a set of simple, domain-independent models that represent the analytical challenges outlined in the introduction section above. Each model was generated as a synthetic OWL ontology with associated instance data and, for all examples, the MCOA method was run with α = 0.15 and ω = 0.01. The ontology, dataset and analysis results for each example are shown in Figure 3.

• **Class overlaps**. Figures 3A and 3B illustrate the two key types of overlaps between non-hierarchically related classes. In Figure 3A, the overlap is due to a single instance being associated with both C2 and C3 (i.e., a multiple annotation overlap). As illustrated by the information content values, analysis based on annotation frequency ignores this overlap and assigns equal weight to C2, C3 and C4. The MCOA method, on the other hand, detects the overlap and divides the impact of the shared instance between C2 and C3 giving these two classes a higher information rank than C4. In Figure 3B, the overlap is due to classes C6 and C7 being associated with multiple parent classes. Because classes C2, C3 and C4 still have equal numbers of instances, they look identical from an information content perspective. The MCOA method also detects this type of overlap and correctly assigns C2 and C4 lower information rank values than C3.

• **Continuously valued data**. Figure 3C contains a variation of the simple ontology from Figure 1A in which some of the instances have been assigned continuous weights. As shown in the figure, a binary assessment of annotation frequency results in uniform information content values for classes C2, C3 and C4. The MCOA approach, because it generates a score that is sensitive to continuous weights, produces the correct differential ranking of C3, C2 and, lastly, C4.

• **Inter-instance relationships**. In Figure 3D, the members of the dataset are connected via inter-instance links with the C2 instances having a balance of in and out links, the C3 instances having net out-links and the C4 instances having net in-links. The MCOA methodology is able to directly integrate the impact of these links and, as shown by the information rank scores, correctly identifies a differential ranking of C4, C2 followed by C3. From the perspective of information content, all three classes appear identical.

• **Semantic distance**. Figure 3E provides a trivial example of semantic distance. Because class C2 is the only child of class C1, it is indistinguishable from an information content perspective. The information rank measure, through the random jump parameter α, reflects the relative semantic distance between the classes, with more specific classes given a higher weight. In this case, the MCOA method correctly assigned C2 a lower information rank than its parent C1.

• **Sparse data**. Figure 3F shows a simple example of a sparse dataset in which one of the classes, C4, lacks associated instances. The MCOA approach, when used with a non-zero α and non-zero ω, supports smoothing of sparse datasets through a form of prior probability weighting resulting from the uniform distribution of random jump probability. As shown in the example, this form of smoothing gives C4 a low, but non-zero, steady state probability and correspondingly high relative information rank.

### Results of GO Enrichment Analysis of Simulated Data

**Figure 4:**
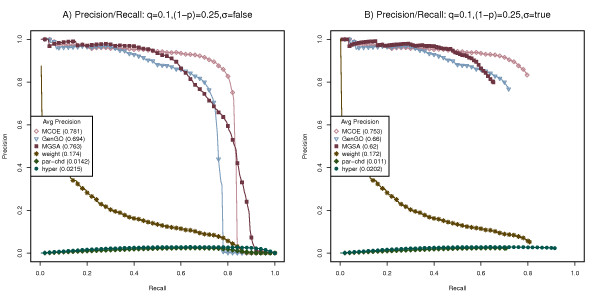
**Benchmarking on simulated Escherichia coli data sets**. Performance of MCOA, MGSA, GenGO, weight, parent-child union and hypergeometric methods on simulated Escherichia coli data sets created with false positive rate (q) of 0.1, false negative rate (1-p) of 0.25. (A) Precision/recall statistics are computed using all categories. (B) Precision/recall statistics are computed using only significantly enriched categories.

**Figure 5:**
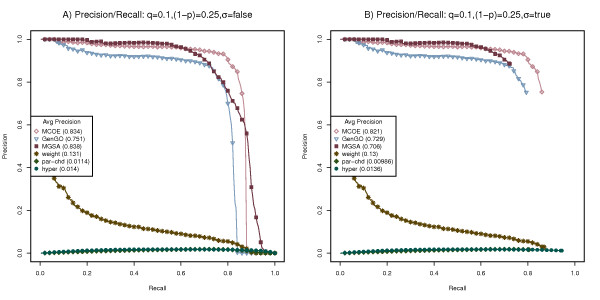
**Benchmarking on simulated Drosophila melanogaster data sets**. Performance of MCOA, MGSA, GenGO, weight, parent-child union and hypergeometric methods on simulated Drosophila melanogaster data sets created with false positive rate (q) of 0.1, false negative rate (1-p) of 0.25. (A) Precision/recall statistics are computed using all categories. (B) Precision/recall statistics are computed using only significantly enriched categories.

**Figure 6:**
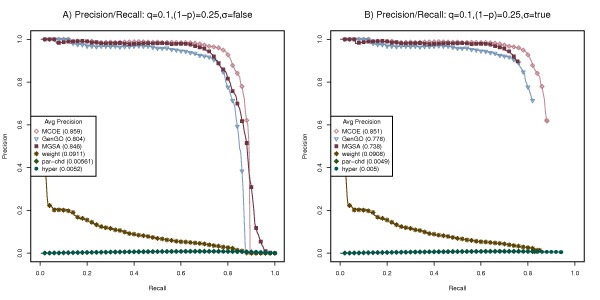
**Benchmarking on simulated Homo sapiens data sets**. Performance of MCOA, MGSA, GenGO, weight, parent-child union and hypergeometric methods on simulated Homo sapiens data sets created with false positive rate (q) of 0.1, false negative rate (1-p) of 0.25. (A) Precision/recall statistics are computed using all categories. (B) Precision/recall statistics are computed using only significantly enriched categories.

Using the benchmarking process outlined above, we tested MCOA enrichment analysis and the other state-of-the-art methods on simulated Escherichia coli, Drosophila melanogaster and Homo sapiens datasets. Figures 4, 5 and 6 display performance/recall curves for datasets generated for each of these species using a false positive rate (q) or 0.1, a false negative rate (1-p) of 0.25, a β of 0.5 and a variable enrichment threshold (σ). Results for four additional false positive and false negative configurations are contained in Additional Files 2,3,4,5,6,7,8,9,10,11,12 and 13 and relative execution time statistics are contained in Additional File 14. For each species and combination of false negative and false positive rates, 500 simulated gene lists were created and the performance of each analysis method was measured using average precision or area under the precision/recall curve.

As the precision/recall curves in Figures 4, 5 and 6 show, the performance of the MEA methods MCOA, GenGO and MGSA dominate the comparable results of the weight, parent-child union and hypergeometric methods for all species and all parameter configurations.

When precision/recall metrics are calculated irrespective of enrichment values, as show in Figures 4A, 5A and 6A, the MCOA method performs measurably better than GenGO for all species, slightly better than MGSA on E. coli and Homo sapiens and on par with MGSA for Drosophila (average precision values for MCOA of 0.781, 0.834 and 0.859 on E. coli, Drosophila and Homo sapiens compared to 0.694, 0.751 and 0.804 for GenGO and 0.763, 0.838 and 0.846 for MGSA). Figures 4B, 5B and 6B show these same results with only statistically significantly enriched GO categories counted as positives for precision/recall statistics. When enrichment significance is considered during precision/recall calculations, the performance edge of the MGSA method disappears and MCOA becomes the clearly superior approach (average precision values for MCOA of 0.753, 0.821 and 0.851 on E. coli, Drosophila and Homo sapiens compared to 0.66, 0.729 and 0.778 for GenGO and 0.62, 0.706 and 0.738 for MGSA). Although p-value and marginal posterior probability thresholds are not directly comparable and a lower threshold for MGSA could plausibly be selected, which would narrow the average performance delta, any reasonable marginal probability threshold would still give MCOA a measurable performance delta over MGSA.

Overall, the MCOA method provides superior enrichment performance across a range of species and experimental parameters. It is important to note that these benchmarking tests, in order to support comparison against other state-of-the-art methods, only reflect performance on data sets that exercise the class overlap and semantic distance challenges. On datasets that incorporate continuous data values, inter-instance relationships, non-hierarchical class relationships or sparse data, the relative advantage of the MCOA method should be even more significant.

### Results of GO Enrichment Analysis of Parkinson's Gene Expression Data

**Figure 7:**
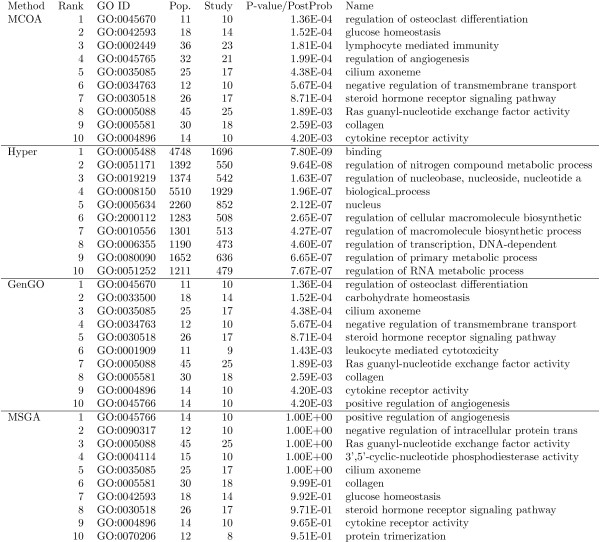
**Analysis of Parkinson's gene expression data from GEO GDS3129**. GO enrichment results on significantly differentially enriched genes in Parkinson's postmortem brain tissue (GEO dataset GDS3129). The top 10 GO terms generated by MCOA, the standard hypergeometric method, GenGO and MGSA are shown for comparison. GO terms are ranked by uncorrected p-value for MCOA, GenGO and hypergeometric and by marginal posterior probability for MGSA. See Additional Files 15, 16, 17 and 18 for complete results.

The top ten enriched GO terms returned by MCOA, hypergeometric, MGSA and GenGO are listed in Figure 7 (enrichment ranking is by uncorrected p-value for MCOA, hypergeometric and GenGO and marginal posterior probability for MGSA; see Additional Files 15, 16, 17 and 18 for complete analysis results). As shown in this figure, all of the top results returned by MCOA are specific, non-overlapping and associated with recently published findings linking the associated biological process, molecular function or cellular component to Parkinson's disease. The top result, *regulation of osteoclast differentiation*, is supported by research linking Parkinson's disease with low bone density/osteoporosis [49,50] as well as the finding of rheumatoid arthritis as a comorbidity [51]. The second result, *glucose homeostasis*, is supported by the link between Parkinson's disease and cortical hypometabolism [52,53] as well as the association between insulin gylcation, glucose homeostasis and Parkinson's [54]. The third result, *lymphocyte mediated immunity*, is supported by research that links neurodegeneration in a mouse model of Parkinson's with the presence of CD4+ lymphocytes in the brain [55]. Similar supporting research is present for the other top ten results (see Additional File 19 for a complete discussion).

The top GO terms returned by the standard hypergeometric method are all at a very high level in the GO tree (the forth ranked result is the root biological process) and a number of terms are redundant due to hierarchical overlap. Although both GenGO and MGSA generate results that are generally similar in content and specificity to those returned by MCOA, a close inspection reveals important differences impacting result quality and utility to experimental scientists. The second term in the GenGO results, *carbohydrate homeostasis*, receives all relevant experimental annotations from the single child *glucose homeostasis. Glucose homeostasis *should therefore be flagged for enrichment instead of *carbohydrate homeostasis*. Because the MCOA regularization term penalizes semantic distance, it correctly ranks *glucose homeostasis *above *carbohydrate homeostasis*. GenGO also fails to return GO term *lymphocyte mediated immunity *in the top ten results and instead identifies the nearby, but less significantly enriched and more general, term *leukocyte mediated cytotoxicity *(*leukocyte mediated cytotoxicity *is a sibling of *leukocyte mediated immunity *which is parent of *lymphocyte mediated immunity*). In this case, all nine differentially expressed genes annotated to *leukocyte medidated cytotoxicity *are also annotated to *lymphocyte mediated immunity*. Because MCOA divides the contribution of a gene between all annotated terms, the more granular *lymphocyte mediated immunity *with some direct gene annotations is preferred over *leukocyte mediated cytotoxicity*. GenGO also includes the overly specific *positive regulation of angiogensis *rather than parent *regulation of angiogensis*. The parent is more appropriate since the other two children (*negative regulation of angiogenesis *and *regulation of cell migration involved in sprouting angiogenesis *are also enriched leading to a much more significant enrichment p-value for *regulation of angiogenesis *(.000199) vs. *positive regulation of angiogenesis *(.0042)). MCOA correctly identifies *regulation of angiogenesis *in the top ten results.

The results returned by the MGSA method have similar issues, when compared to MCOA, as the GenGO results (e.g., MGSA also fails to identify *lymphocyte mediated immunity *and ranks *positive regulation of angiogenesis *in the top ten rather than *regulation of angiogenesis*). In terms of utility for users, however, a more significant difference between MCOA and MGSA relates to MGSA's use of marginal posterior probabilities and the impact these probabilities have on ranking and interpretation of enrichment results. Although both MCOA and MGSA identify many similar GO terms in the top rankings, the marginal posterior probability rankings of MGSA can differ substantially from what is achieved when hypergeometric p-values are used on the terms that optimize the objective function. We believe that the use of hypergeometric p-values by MCOA and GenGO leads to a top set of rankings whose relative order and statistical significance is more easily interpretable by scientists.

## Discussion

### The Challenge of Biological Complexity

Ontology-based data analysis methods such as enrichment analysis and semantic similarity clustering have become critical tools for processing the experimental results of modern biomedical science. Without the abstract lens of classifications such as GO and KEGG, the large gene and protein lists generated by molecular biological research would be difficult to analyze manually and almost impossible to compare meaningfully across experimental populations or species. Despite the important role that these methods play in interpreting and guiding biomedical research, their utility has been hampered by the limitations of traditional analytical methods to handle the complex interdependencies present in real biomedical data and associated data models. The members of real biological datasets do not cleanly sort into independent classes but instead group into complex collections of nested and overlapping categories, with direct relationships between dataset members and a mixture of continuous and categorical data values.

Tackling this complexity requires methods that perform a global, rather than local, analysis of the ontology and dataset to capture the full range of structural interdependencies and data values. Although recent methods in the GSEA and MEA categories have made notable advances in this area, specifically in addressing class overlaps and continuously valued data, the interesting features of many biological datasets remain inaccessible to analytical tools. To help address the challenge of biological complexity, we developed the MCOA method as a network analytic framework capable of addressing the class overlap and continuously valued data challenges targeted by MEA and GSEA methods as well as supporting continuous relationship values, inter-instance relations, non-hierarchical class relations, semantic distance and sparse data.

### Advantages of the MCOA Markov Chain Model

Underlying the MCOA method's analytical behaviour and its ability to successfully detect structural complexity is the method employed for building a Markov chain model and computing steady state probabilities. Several features of the MCOA Markov chain model are critical to its functionality:

• **Assignment of probabilistic weight per instance rather than per annotation**. Under the MCOA Markov chain model, the weight for each dataset instance is divided among all of the classes to which the instance is annotated. This weight is initially divided among all direct annotations of the instance and, as it propagates through the Markov chain, consolidates in an increasingly smaller number of classes until the entire instance weight is concentrated at the root. The MCOA approach contrasts with the annotation frequency approach in which the full instance weight is assigned to each annotated class with the effect that instances shared by many classes contribute the same weight as instances annotated to only a single class. MCOA uses the differential contribution of instances with a large number of class annotations and those with small number of annotations to help detect class overlaps resulting from multiple annotations and multiple parents.

• **Flexible relationships**. Traditional analysis methods only model hierarchical class relationships and class-to-instance annotations. Some methods, such as GenGO and MGSA, ignore most hierarchical information by analyzing a collapsed representation of the ontology graph. The MCOA method, in contrast, analyzes the full ontology and dataset network and can additionally handle relationships, such as inter-instance relationships and non-hierarchical relationships between classes, that are important for modelling real biomedical data but are not directly supported by existing MEA approaches.

• **Semantic distance computation**. The use of a random jump parameter allows semantic distance to be quantified and hierarchical overlaps to be detected, since the amount of transferred rank naturally decays with each transition up the ontology hierarchy. Although semantic distance is captured at some level by enrichment methods such as elim and weight, it is ignored by the more recent MEA approaches GenGO and MGSA as well as by techniques in the GSEA category.

• **Continuous values for instances, classes and relationships**. A non-uniform distribution of random jump probabilities can be used in the MCOA method to mirror differential class and instance weights. The Markov chain model also enables continuous values to be applied to inter-class, class-to-instance or inter-instance relationships. With existing state-of-the-art analysis methods, support for continuous data values is usually limited to dataset instances.

• **Prior weighting**. The non-uniform distribution of random jump probability also allows the MCOA method to apply any desired prior probability distribution to achieve smoothing of sparse data or to align with a Bayesian analysis approach.

### MCOA for Enrichment Analysis

We chose enrichment analysis as the context in which to explore and validate the functionality of the MCOA method. In developing and benchmarking a MCOA-based enrichment analysis approach, we aimed to create an enrichment tool with the best performance among existing state-of-the-art methods on simulated datasets created to highlight the complexities encountered in real biomedical data. We also aimed to create a practical methodology capable of generating enrichment results on real data sets that are specific, non-overlapping and of high utility to experimental biologists. The superior performance achieved by the MCOA enrichment analysis approach can be understood in terms of the kinds of type I and type II errors encountered by the other generative methods (GenGO and MGSA) but avoided by MCOA.

In this context, type I, or false positive, errors represent cases where an enrichment method incorrectly identifies a non-active category as enriched. There were two varieties of type I errors commonly made by the other generative methods that were avoided by MCOA:

• **Incorrectly flagging non-active categories that are more general than an active category**. In these cases, the more general category appears enriched because it is inheriting all of the annotations from the active category along with a significant number of additional annotations enabled due to noise. MCOA is able to correctly ignore these categories because the contributions from the active category are discounted due to both semantic distance and overlaps with other classes. GenGO and MGSA, because they collapse the ontology graph and give each annotation equal weight regardless of the number of annotations, do not discount the contributions from the active category and incorrectly flag the more general category as enriched.

• **Incorrectly flagging non-active categories that are not hierarchically related to an active category, have a small number of associated genes and few or no direct annotations**. In these cases, the non-active category appears enriched due to noise. Because these categories have few annotated genes and almost no directly annotated genes, MCOA assigns the category a low steady-state probability and does not include it in the set of significantly enriched categories. Because the other generative methods assign weight per annotation and ignore semantic distance, they give the category an incorrectly high weight and mark it as enriched.

Type II, or false negative, errors represent cases where an enrichment method fails to identify an active category as enriched. In our experiments, the other generative methods commonly failed to identify as enriched active categories that had a small number of directly annotated genes. When analyzed by MCOA, these categories have a higher relative steady-state probability due to both the lack of a semantic distance discount for the direct annotations and the fact that direct annotations will not have overlaps due to multiple parents. Because of this higher relative steady-state probability, MCOA is able to successfully mark these categories as enriched. GenGO and MGSA, on the other hand, do not give any special weight to the direct annotations and therefore fail to detect the relatively higher enrichment of these categories.

### MCOA Limitations

Limitations of the MCOA method and MCOA-based enrichment analysis include a comparatively high computational complexity relative to other methods (see Additional File 14 for execution time statistics), reliance on the GenGO approach for objective function optimization through greedy search and sensitivity to the specified values of the false positive and false negative rates (variation in the p and q values can dramatically impact the number of GO terms that optimize the objective function for a given data set).

### Other MCOA Applications

Although the discussion and examples in this paper have primarily focused on the use of the MCOA method for enrichment analysis, the same general approach can be used to support other ontology-based analysis applications, such as:

• **Semantic similarity clustering**: Semantic similarity algorithms that use the information content of classes (e.g., Resnik [56]) can be modified to use information rank instead.

• **Ontology evaluation**: Similar to the modification of semantic similarity algorithms, existing statistical ontology evaluation approaches that leverage information content (e.g., Alterovitz *et al *[4]) can be modified to use the MCOA-based information rank. The underlying steady state probabilities can also be employed to weight class-specific evaluation metrics when computing overall ontology evaluation scores.

• **Ontology-driven information retrieval**. If the Markov chain is created such that state transitions flow from classes in the ontology to instances, instance-level steady-state probability values can be computed that quantify the importance of each instance relative to the classes in the ontology.

• **Ontology comparative analysis**. If state transitions flow from the classes, through a set of associated instances and into the classes in another ontology, it becomes possible to use the MCOA method to quantify the importance of one set of classes relative to another set of classes based on the annotations of a common dataset. Comparative analysis of multiple ontologies can also be enabled through non-hierarchical relationships between the classes in one ontology and the classes in another ontology.

## Conclusion

Biomedical ontologies have become increasingly critical for the analysis, retrieval and integration of large and complex datasets. Of particular importance are applications, such as enrichment analysis, that measure the importance of ontology classes relative to a collection of domain data. Current analysis methods, however, remain limited in their ability to detect and accurately quantify a range of complex structural features at the ontological and dataset levels. To help address these challenges, we developed the Markov Chain Ontology Analysis (MCOA) methodology and used this method to create the MCOA extension of the GenGO enrichment analysis approach.

The core MCOA method can detect structural features including class overlaps, continuous data values, relationships between data instances, semantic distance and sparse data that are difficult to detect using standard annotation frequency analysis. In benchmarking studies on simulated Escherichia coli, Drosophila melanogaster and Homo sapiens datasets highlighting the complexities of biomedical data, the MCOA enrichment analysis method provides the best performance of comparable state-of the-art Gene Ontology enrichment methods. On real experimental data, MCOA has been shown to provide specific, non-redundant and scientifically valid results.

As next steps, we plan to conduct benchmarking on datasets that capture a wider range of analytical challenges (e.g., continuous weights and inter-instance relationships), use the MCOA enrichment analysis method to analyze and interpret additional experimental data sets, and perform enrichment against ontologies other than the Gene Ontology. We also plan to explore the use of the MCOA information rank value for applications that have traditionally employed information content, such as ontology evaluation and semantic similarity clustering.

An implementation of the MCOA-based enrichment analysis tool can be accessed at the project homepage [48].

## Authors' contributions

HRF conceived of the methodology, implemented the MCOA algorithm and MCOA enrichment analysis method, performed the reported data analysis and drafted the manuscript. ATM participated in the development of the methodology, selection and analysis of use cases and revision of the manuscript. Both HRF and ATM have read and approve the final manuscript.

## Supplementary Material

Additional File 1 **Gene Ontology term overlap statistics with Homo sapiens annotations**. Click here for file

Additional File 2 **Benchmarking results on simulated Escherichia coli data sets for false positive rate (q) of 0.01 and false negative rate (1-p) of 0.1**. Click here for file

Additional File 3 **Benchmarking results on simulated Drosophila Melanogaster data sets for false positive rate (q) of 0.01 and false negative rate (1-p) of 0.1**. Click here for file

Additional File 4 **Benchmarking results on simulated Homo sapiens data sets for false positive rate (q) of 0.01 and false negative rate (1-p) of 0.1**. Click here for file

Additional File 5 **Benchmarking results on simulated Escherichia coli data sets for false positive rate (q) of 0.4 and false negative rate (1-p) of 0.25**. Click here for file

Additional File 6 **Benchmarking results on simulated Drosophila Melanogaster data sets for false positive rate (q) of 0.4 and false negative rate (1-p) of 0.25**. Click here for file

Additional File 7 **Benchmarking results on simulated Homo sapiens data sets for false positive rate (q) of 0.4 and false negative rate (1-p) of 0.25**. Click here for file

Additional File 8 **Benchmarking results on simulated Escherichia coli data sets for false positive rate (q) of 0.1 and false negative rate (1-p) of 0.1**. Click here for file

Additional File 9 **Benchmarking results on simulated Drosophila Melanogaster data sets for false positive rate (q) of 0.1 and false negative rate (1-p) of 0.1**. Click here for file

Additional File 10 **Benchmarking results on simulated Homo sapiens data sets for false positive rate (q) of 0.1 and false negative rate (1-p) of 0.1**. Click here for file

Additional File 11 **Benchmarking results on simulated Escherichia coli data sets for false positive rate (q) of 0.1 and false negative rate (1-p) of 0.4**. Click here for file

Additional File 12 **Benchmarking results on simulated Drosophila Melanogaster data sets for false positive rate (q) of 0.1 and false negative rate (1-p) of 0.4**. Click here for file

Additional File 13 **Benchmarking results on simulated Homo sapiens data sets for false positive rate (q) of 0.1 and false negative rate (1-p) of 0.4**. Click here for file

Additional File 14 **Relative execution time statistics on simulated Homo sapiens data**. Click here for file

Additional File 15 **Full analysis results for MCOA on GEO dataset GDS3129**. Click here for file

Additional File 16 **Full analysis results for hypergeometric method on GEO dataset GDS3129**. Click here for file

Additional File 17 **Full analysis results for MGSA method on GEO dataset GDS3129**. Click here for file

Additional File 18 **Full analysis results for GenGO method on GEO dataset GDS3129**. Click here for file

Additional File 19 **Research linking top ten GO terms returned by MCOA on GEO dataset GDS3129 and Parkinson's disease**. Click here for file

## References

[B1] Bodenreider O Mitchell JA McCray AT Biomedical ontologies Pac Symp Biocomput 2005 76 78 15759615 10.1142/9789812704856_0016 PMC4300097

[B2] Huang DW Sherman BT Lempicki RA Bioinformatics enrichment tools: paths toward the comprehensive functional analysis of large gene lists Nucleic Acids Research 2009 37 1 13 10.1093/nar/gkn923 19033363 PMC2615629

[B3] Pesquita C Faria D Falcão AO Lord P Couto FM Semantic similarity in biomedical ontologies PLoS Comput Biol 2009 5 e1000443 10.1371/journal.pcbi.1000443 19649320 PMC2712090

[B4] Alterovitz G Xiang M Hill DP Lomax J Liu J Cherkassky M Dreyfuss J Mungall C Harris MA Dolan ME Blake JA Ramoni MF Ontology engineering Nat Biotechnol 2010 28 128 130 10.1038/nbt0210-128 20139945 PMC4829499

[B5] Ashburner M Ball CA Blake JA Botstein D Butler H Cherry JM Davis AP Dolinski K Dwight SS Eppig JT Harris MA Hill DP Issel-Tarver L Kasarskis A Lewis S Matese JC Richardson JE Ringwald M Rubin GM Sherlock G Gene Ontology: tool for the unification of biology Nat Genet 2000 25 25 29 10.1038/75556 10802651 PMC3037419

[B6] Kanehisa M Goto S KEGG: kyoto encyclopedia of genes and genomes Nucleic Acids Res 2000 28 27 30 10.1093/nar/28.1.27 10592173 PMC102409

[B7] Tirrell R Evani U Berman AE Mooney SD Musen MA Shah NH An ontology-neutral framework for enrichment analysis AMIA Annu Symp Proc 2010 2010 797 801 21347088 PMC3041299

[B8] Noy NF Shah NH Whetzel PL Dai B Dorf M Griffith N Jonquet C Rubin DL Storey M-A Chute CG Musen MA BioPortal: ontologies and integrated data resources at the click of a mouse Nucleic Acids Research 2009 37 W170 W173 10.1093/nar/gkp440 19483092 PMC2703982

[B9] Shah NH Jonquet C Chiang AP Butte AJ Chen R Musen MA Ontology-driven indexing of public datasets for translational bioinformatics BMC Bioinformatics 2009 10 Suppl 2 S1 10.1186/1471-2105-10-S2-S1 19208184 PMC2646250

[B10] Subramanian A Tamayo P Mootha VK Mukherjee S Ebert BL Gillette MA Paulovich A Pomeroy SL Golub TR Lander ES Mesirov JP Gene set enrichment analysis: A knowledge-based approach for interpreting genome-wide expression profiles Proc Natl Acad Sci USA 2005 102 15545 15550 10.1073/pnas.0506580102 16199517 PMC1239896

[B11] Newton A Quintana FA Den JA Sengupta S Ahlquist P Chile C Random-Set Methods Identify Distinct Aspects of the Enrichment Signal in Gene-Set Analysis," The Annals of Applied Statistics 2007

[B12] Sartor MA Leikauf GD Medvedovic M LRpath: a logistic regression approach for identifying enriched biological groups in gene expression data Bioinformatics 2009 25 211 217 10.1093/bioinformatics/btn592 19038984 PMC2639007

[B13] Wang J Huang Q Liu Z-P Wang Y Wu L-Y Chen L Zhang X-S NOA: a novel Network Ontology Analysis method Nucleic Acids Research 2011 39 e87 10.1093/nar/gkr251 21543451 PMC3141273

[B14] Glaab E Baudot A Krasnogor N Valencia A TopoGSA: network topological gene set analysis Bioinformatics 2010 26 1271 1272 10.1093/bioinformatics/btq131 20335277 PMC2859135

[B15] Lu Y Rosenfeld R Simon I Nau GJ Bar-Joseph Z A probabilistic generative model for GO enrichment analysis Nucleic Acids Research 2008 36 e109 10.1093/nar/gkn434 18676451 PMC2553574

[B16] Bauer S Gagneur J Robinson PN Going Bayesian: model-based gene set analysis of genome-scale data Nucleic Acids Research 2010 38 3523 3532 10.1093/nar/gkq045 20172960 PMC2887944

[B17] Bauer S Robinson PN Gagneur J Model-based gene set analysis for Bioconductor Bioinformatics 2011 27 1882 1883 10.1093/bioinformatics/btr296 21561920 PMC3117381

[B18] Wang J Zhou X Zhu J Zhou C Guo Z Revealing and avoiding bias in semantic similarity scores for protein pairs BMC Bioinformatics 2010 11 290 10.1186/1471-2105-11-290 20509916 PMC2903568

[B19] Brin S Page L The anatomy of a large-scale hypertextual Web search engine Computer Networks and ISDN Systems 1998 30 Amsterdam, The Netherlands, The Netherlands: Elsevier Science Publishers B.V. 107 117 10.1016/S0169-7552(98)00110-X

[B20] Grossmann S Bauer S Robinson PN Vingron M Improved detection of overrepresentation of Gene-Ontology annotations with parent child analysis Bioinformatics 2007 23 3024 3031 10.1093/bioinformatics/btm440 17848398

[B21] Alexa A Rahnenführer J Lengauer T Improved scoring of functional groups from gene expression data by decorrelating GO graph structure Bioinformatics 2006 22 1600 1607 10.1093/bioinformatics/btl140 16606683

[B22] Vêncio RZN Shmulevich I ProbCD: enrichment analysis accounting for categorization uncertainty BMC Bioinformatics 2007 8 383 10.1186/1471-2105-8-383 17935624 PMC2169266

[B23] Pavlopoulos GA Secrier M Moschopoulos CN Soldatos TG Kossida S Aerts J Schneider R Bagos PG Using graph theory to analyze biological networks BioData Min 2011 4 10 10.1186/1756-0381-4-10 21527005 PMC3101653

[B24] Almaas E Biological impacts and context of network theory J Exp Biol 2007 210 1548 1558 10.1242/jeb.003731 17449819

[B25] Vidal M Cusick ME Barabási A-L Interactome networks and human disease Cell 2011 144 986 998 10.1016/j.cell.2011.02.016 21414488 PMC3102045

[B26] Vêncio RZN Koide T Gomes SL Pereira CA de B BayGO: Bayesian analysis of ontology term enrichment in microarray data BMC Bioinformatics 2006 7 86 10.1186/1471-2105-7-86 16504085 PMC1440873

[B27] Bade K Benz D Fink A, Lausen B, Seidel W, Ultsch A Evaluation Strategies for Learning Algorithms of Hierarchies Advances in Data Analysis, Data Handling and Business Intelligence 2009 Berlin, Heidelberg: Springer Berlin Heidelberg 83 92

[B28] Cimiano P Ontology Learning and Population from Text: Algorithms, Evaluation and Applications 2006 1 Springer

[B29] Baader F Calvanese D McGuinness D Nardi D Patel-Schneider P The Description Logic Handbook: Theory, Implementation and Applications 2003 Cambridge University Press

[B30] Kemeny JG Snell JL Finite Markov Chains 1960 D. Van Nostrand

[B31] Haveliwala TH Topic-sensitive PageRank Proceedings of the 11th international conference on World Wide Web 2002 New York, NY, USA: ACM 517 526

[B32] Moore JH Asselbergs FW Williams SM Bioinformatics challenges for genome-wide association studies Bioinformatics 2010 26 445 455 10.1093/bioinformatics/btp713 20053841 PMC2820680

[B33] The Gene Ontology http://www.geneontology.org/

[B34] FlyBase Homepage http://flybase.org/

[B35] Gene Ontology Annotation (UniProtKB-GOA) Home Page | EBI http://www.ebi.ac.uk/GOA/

[B36] EcoCyc: Encyclopedia of Escherichia coli K-12 Genes and Metabolism http://ecocyc.org/

[B37] PortEco: portal for E. coli research http://www.ecolihub.org/

[B38] Barrett T Troup DB Wilhite SE Ledoux P Evangelista C Kim IF Tomashevsky M Marshall KA Phillippy KH Sherman PM Muertter RN Holko M Ayanbule O Yefanov A Soboleva A NCBI GEO: archive for functional genomics data sets--10 years on Nucleic Acids Research 2010 39 D1005 D1010 21097893 10.1093/nar/gkq1184 PMC3013736

[B39] Moran LB Duke DC Deprez M Dexter DT Pearce RKB Graeber MB Whole genome expression profiling of the medial and lateral substantia nigra in Parkinson's disease Neurogenetics 2006 7 1 11 10.1007/s10048-005-0020-2 16344956

[B40] Sean D Meltzer PS GEOquery: a bridge between the Gene Expression Omnibus (GEO) and BioConductor Bioinformatics 2007 23 1846 1847 10.1093/bioinformatics/btm254 17496320

[B41] Smyth GK Linear models and empirical bayes methods for assessing differential expression in microarray experiments Stat Appl Genet Mol Biol 2004 3 Article3 10.2202/1544-6115.1027 16646809

[B42] Falcon S Gentleman R Using GOstats to test gene lists for GO term association Bioinformatics 2007 23 257 258 10.1093/bioinformatics/btl567 17098774

[B43] JUNG - Java Universal Network/Graph Framework http://jung.sourceforge.net/index.html

[B44] Math - Commons Math: The Apache Commons Mathematics Library http://commons.apache.org/math/

[B45] Jena Semantic Web Framework http://jena.sourceforge.net/

[B46] OWL Web Ontology Language Reference http://www.w3.org/TR/owl-ref/

[B47] Bauer S Grossmann S Vingron M Robinson PN Ontologizer 2.0--a multifunctional tool for GO term enrichment analysis and data exploration Bioinformatics 2008 24 1650 1651 10.1093/bioinformatics/btn250 18511468

[B48] MCOA Project Homepage http://combo.cbmi.med.harvard.edu/mcoa/

[B49] Gnädinger M Mellinghoff H-U Kaelin-Lang A Parkinson's disease and the bones Swiss Med Wkly 2011 141 w13154 21328097 10.4414/smw.2011.13154

[B50] Invernizzi M Carda S Viscontini GS Cisari C Osteoporosis in Parkinson's disease Parkinsonism Relat Disord 2009 15 339 346 10.1016/j.parkreldis.2009.02.009 19346153

[B51] Gupta M Cheung C-L Hsu Y-H Demissie S Cupples LA Kiel DP Karasik D Identification of homogeneous genetic architecture of multiple genetically correlated traits by block clustering of genome-wide associations J Bone Miner Res 2011 26 1261 1271 10.1002/jbmr.333 21611967 PMC3312758

[B52] Borghammer P Chakravarty M Jonsdottir KY Sato N Matsuda H Ito K Arahata Y Kato T Gjedde A Cortical hypometabolism and hypoperfusion in Parkinson's disease is extensive: probably even at early disease stages Brain Struct Funct 2010 214 303 317 10.1007/s00429-010-0246-0 20361208

[B53] Pappatà S Santangelo G Aarsland D Vicidomini C Longo K Bronnick K Amboni M Erro R Vitale C Caprio MG Pellecchia MT Brunetti A De Michele G Salvatore M Barone P Mild cognitive impairment in drug-naive patients with PD is associated with cerebral hypometabolism Neurology 2011 77 1357 1362 10.1212/WNL.0b013e3182315259 21940621

[B54] Oliveira LMA Lages A Gomes RA Neves H Família C Coelho AV Quintas A Insulin glycation by methylglyoxal results in native-like aggregation and inhibition of fibril formation BMC Biochem 2011 12 41 10.1186/1471-2091-12-41 21819598 PMC3175161

[B55] Brochard V Combadière B Prigent A Laouar Y Perrin A Beray-Berthat V Bonduelle O Alvarez-Fischer D Callebert J Launay J-M Duyckaerts C Flavell RA Hirsch EC Hunot S Infiltration of CD4+ lymphocytes into the brain contributes to neurodegeneration in a mouse model of Parkinson disease J Clin Invest 2009 119 182 192 19104149 10.1172/JCI36470 PMC2613467

[B56] Resnik P Using Information Content to Evaluate Semantic Similarity in a Taxonomyproceedings of the 14th international joint conference on artificial intelligence 1995 448 453

