# Supplementary material for: Markov Chain Ontology Analysis (MCOA)
Source: BMC Bioinformatics. 2012 Feb 3;13:23. doi: 10.1186/1471-2105-13-23 (PMC3329418; doi:10.1186/1471-2105-13-23)
Supplement: Additional File 1 — Gene Ontology term overlap statistics with Homo sapiens annotations. [file 1471-2105-13-23-S1.PDF]

Markov Chain Ontology Analysis - Supplementary Material: Overlaps between Gene Ontology terms based on Homo Sapiens annotations

Overlaps between Gene Ontology terms (GO revision 1.2078, 34,171 total GO terms) based on Homo Sapiens annotations from Go Annotations @ EBI (file revision 1.197; 18,307 gene products with 237,437 annotations to GO categories). Statistics were generated using the Ontologizer OverlapLister tool and capture overlaps based on both GO heirarchy as well as genes with multiple direct annotations.

Descriptive statistics for GO term overlaps:

```
   Min. 1st Qu.  Median    Mean 3rd Qu.    Max. 
      6     186     483    1078    1225   14790 
```

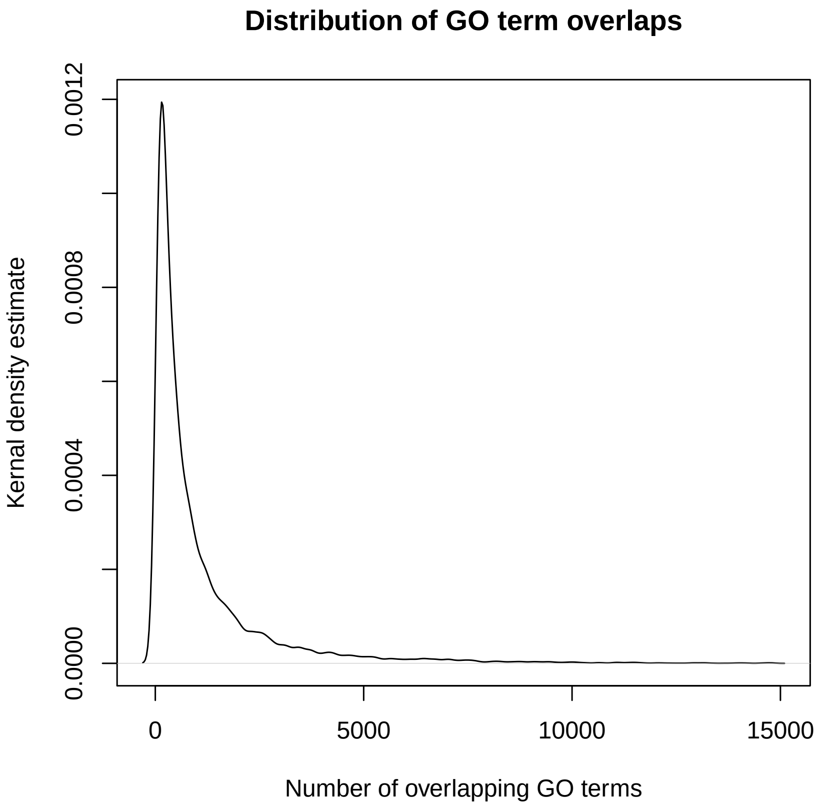
Distribution of GO term overlaps
Kernal density estimate
0.0000
0.0004
0.0008
0.0012
0
5000
10000
15000
Number of overlapping GO terms
