# Supplementary figures and images for: Markov Chain Ontology Analysis (MCOA)
Source: BMC Bioinformatics. 2012 Feb 3;13:23. doi: 10.1186/1471-2105-13-23 (PMC3329418; doi:10.1186/1471-2105-13-23)

A) Precision/Recall:  $q=0.01, (1-p)=0.1, \sigma=\text{false}$

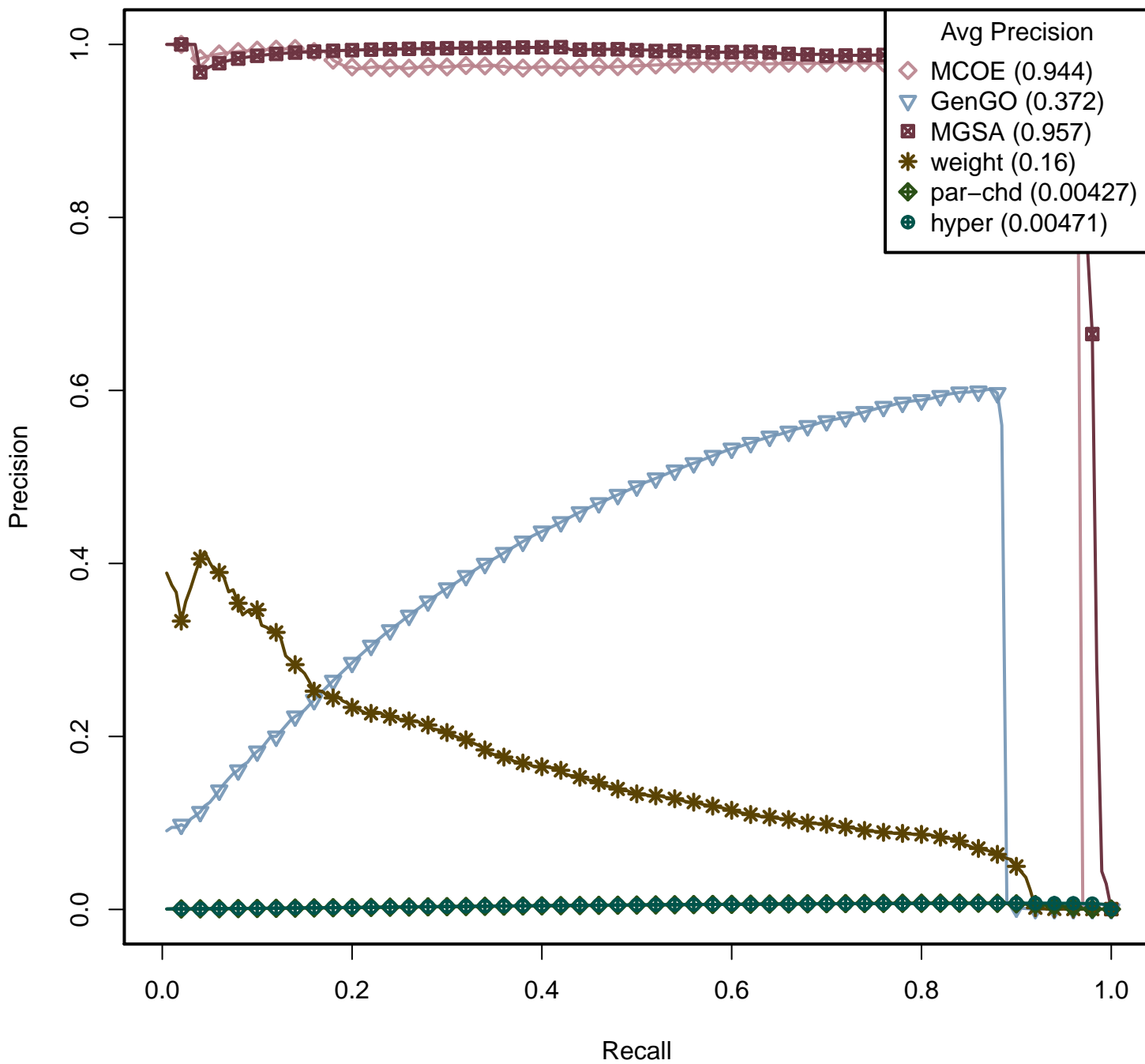

B) Precision/Recall:  $q=0.01, (1-p)=0.1, \sigma=\text{true}$

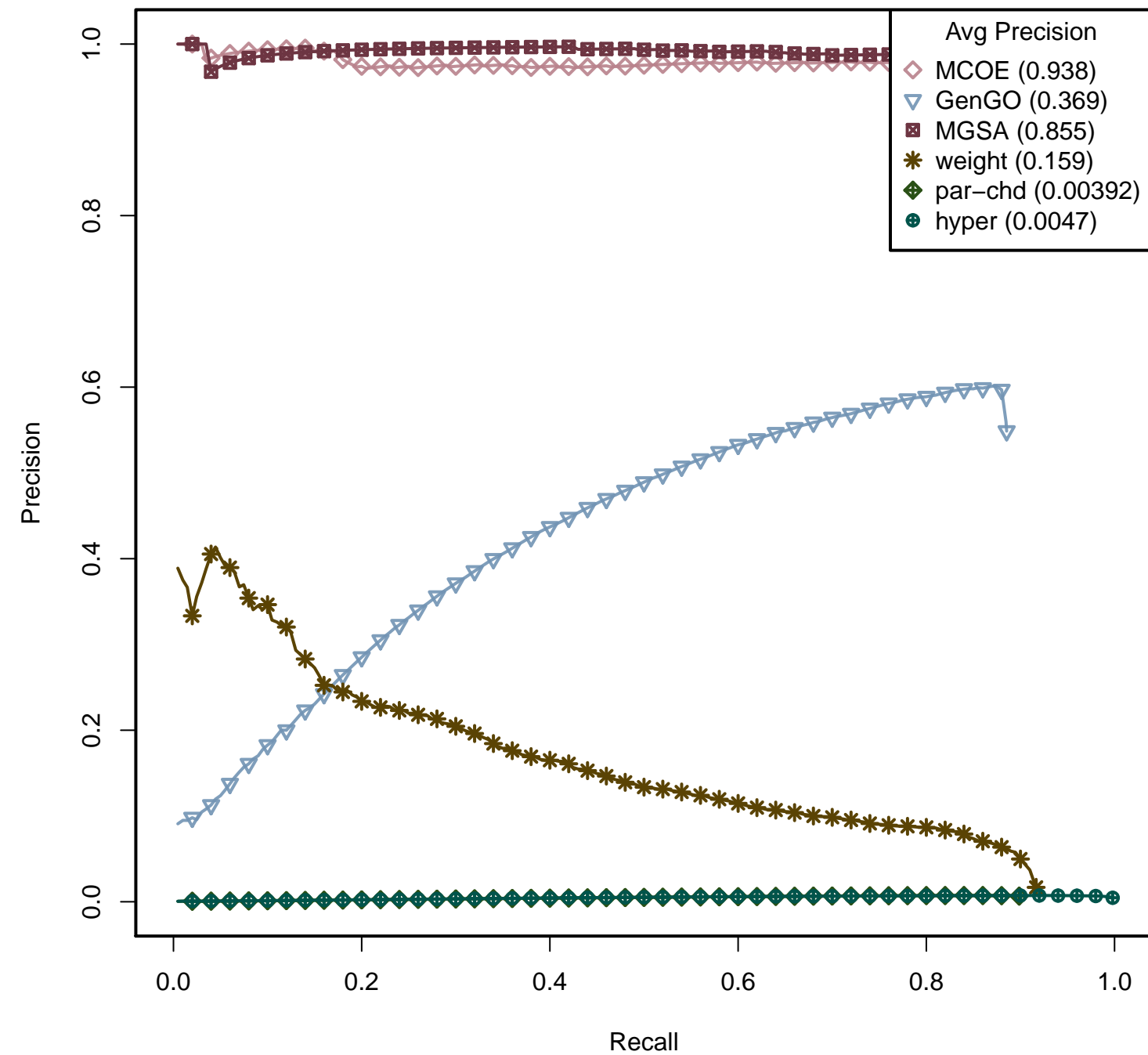

Supplement: Additional File 2 — Benchmarking results on simulated Escherichia coli data sets for false positive rate (q) of 0.01 and false negative rate (1-p) of 0.1. [file 1471-2105-13-23-S2.PDF]

A) Precision/Recall:  $q=0.01, (1-p)=0.1, \sigma=\text{false}$

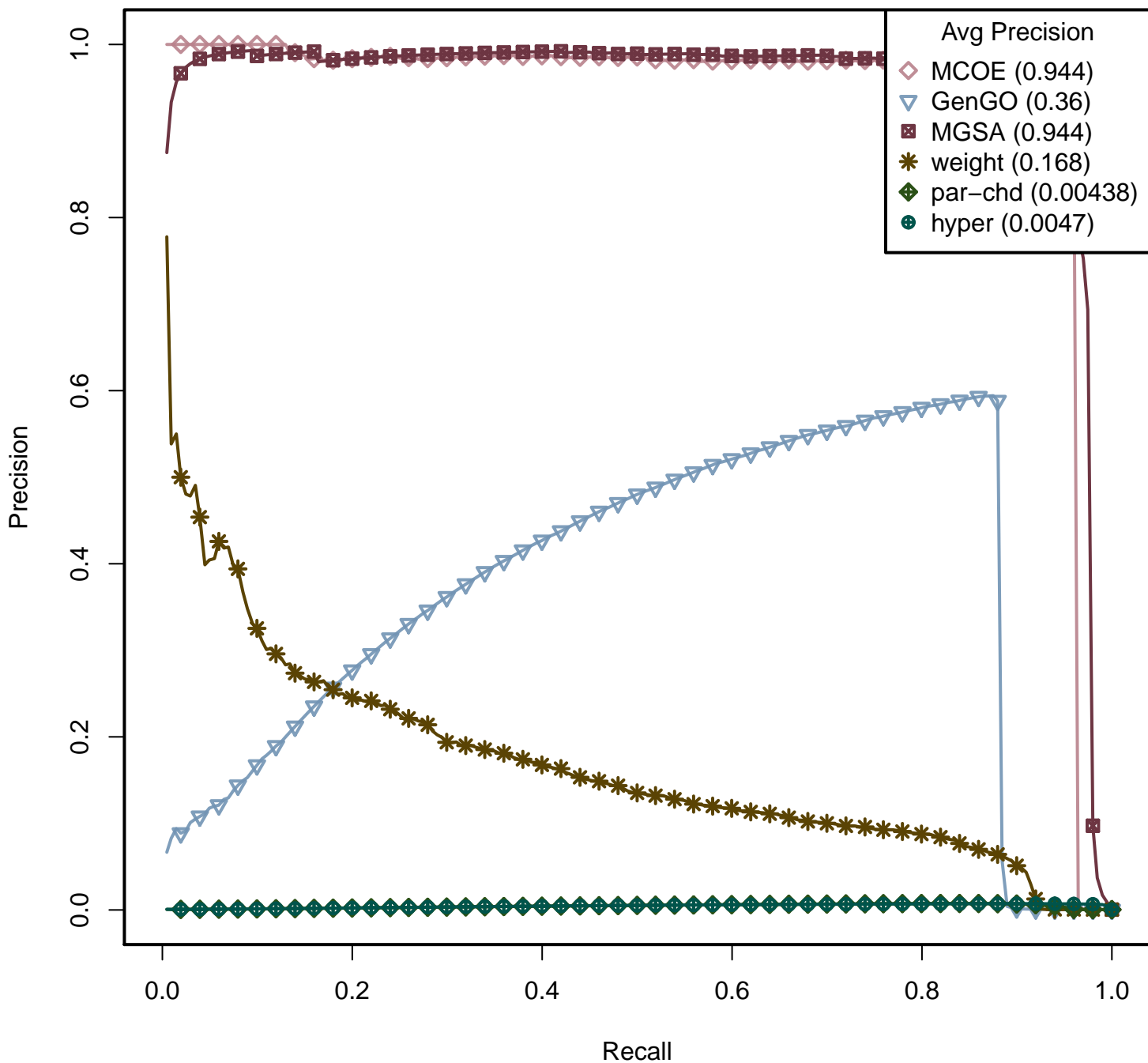

B) Precision/Recall:  $q=0.01, (1-p)=0.1, \sigma=\text{true}$

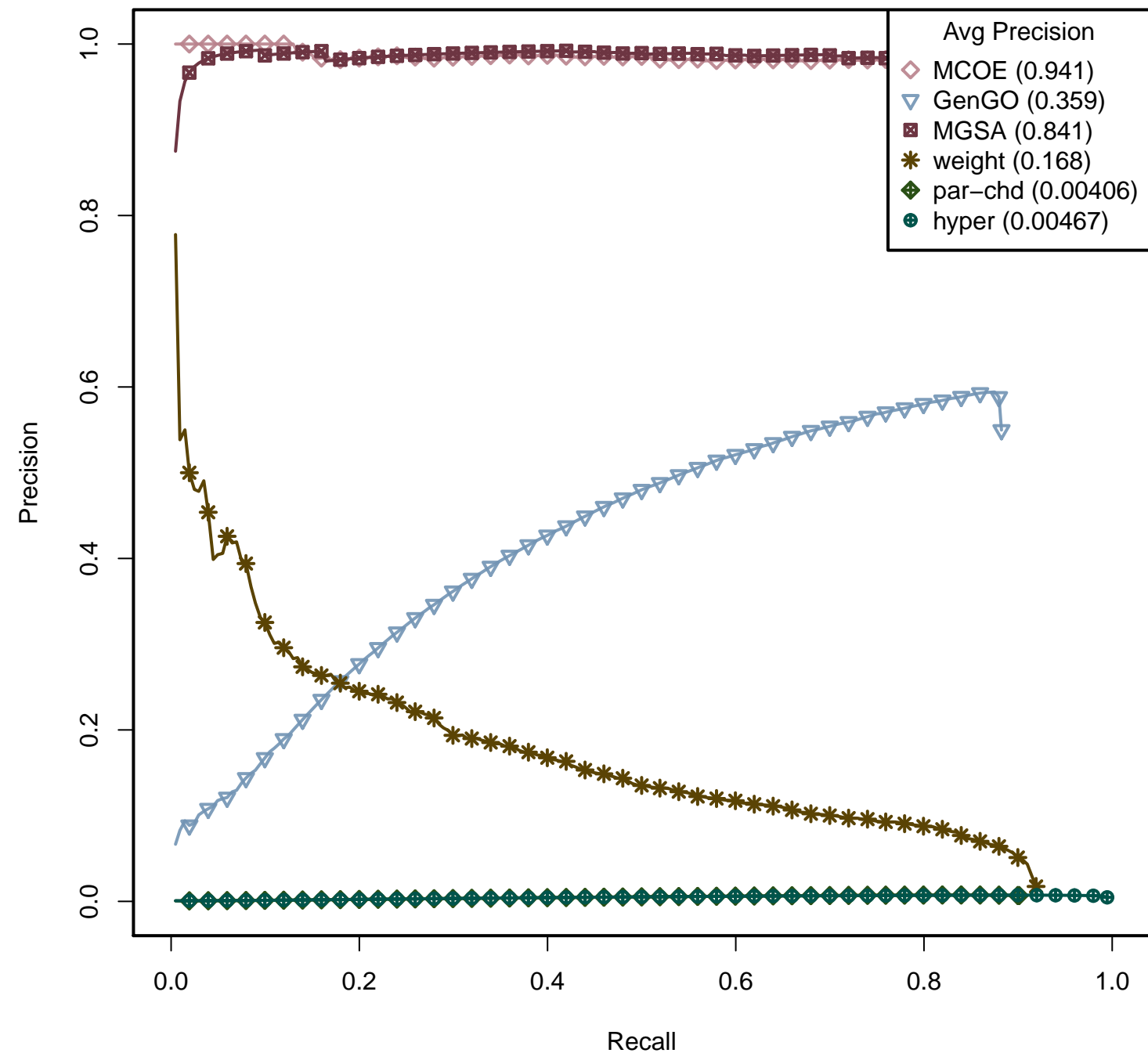

Supplement: Additional File 3 — Benchmarking results on simulated Drosophila Melanogaster data sets for false positive rate (q) of 0.01 and false negative rate (1-p) of 0.1. [file 1471-2105-13-23-S3.PDF]

A) Precision/Recall:  $q=0.01, (1-p)=0.1, \sigma=\text{false}$

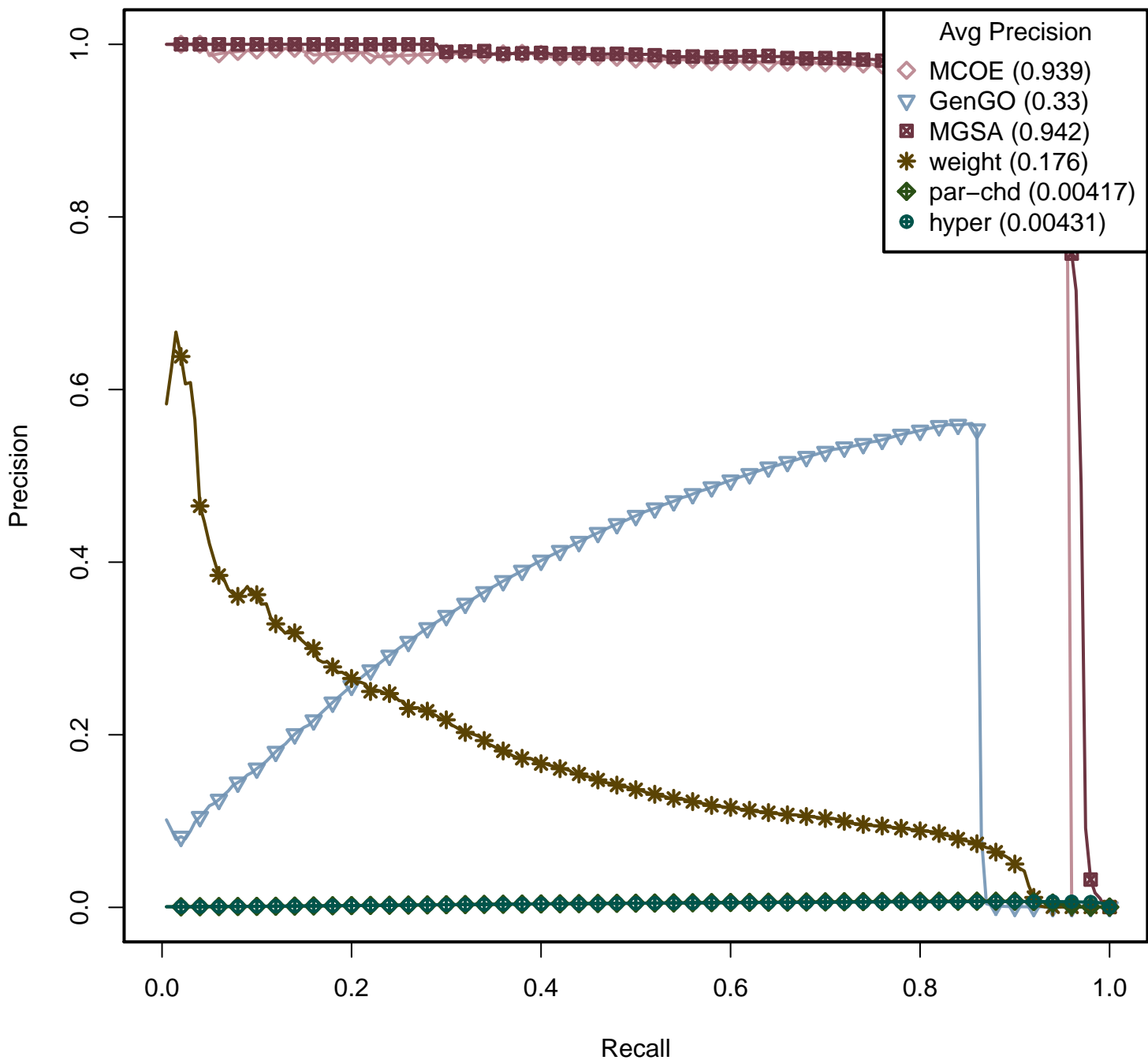

B) Precision/Recall:  $q=0.01, (1-p)=0.1, \sigma=\text{true}$

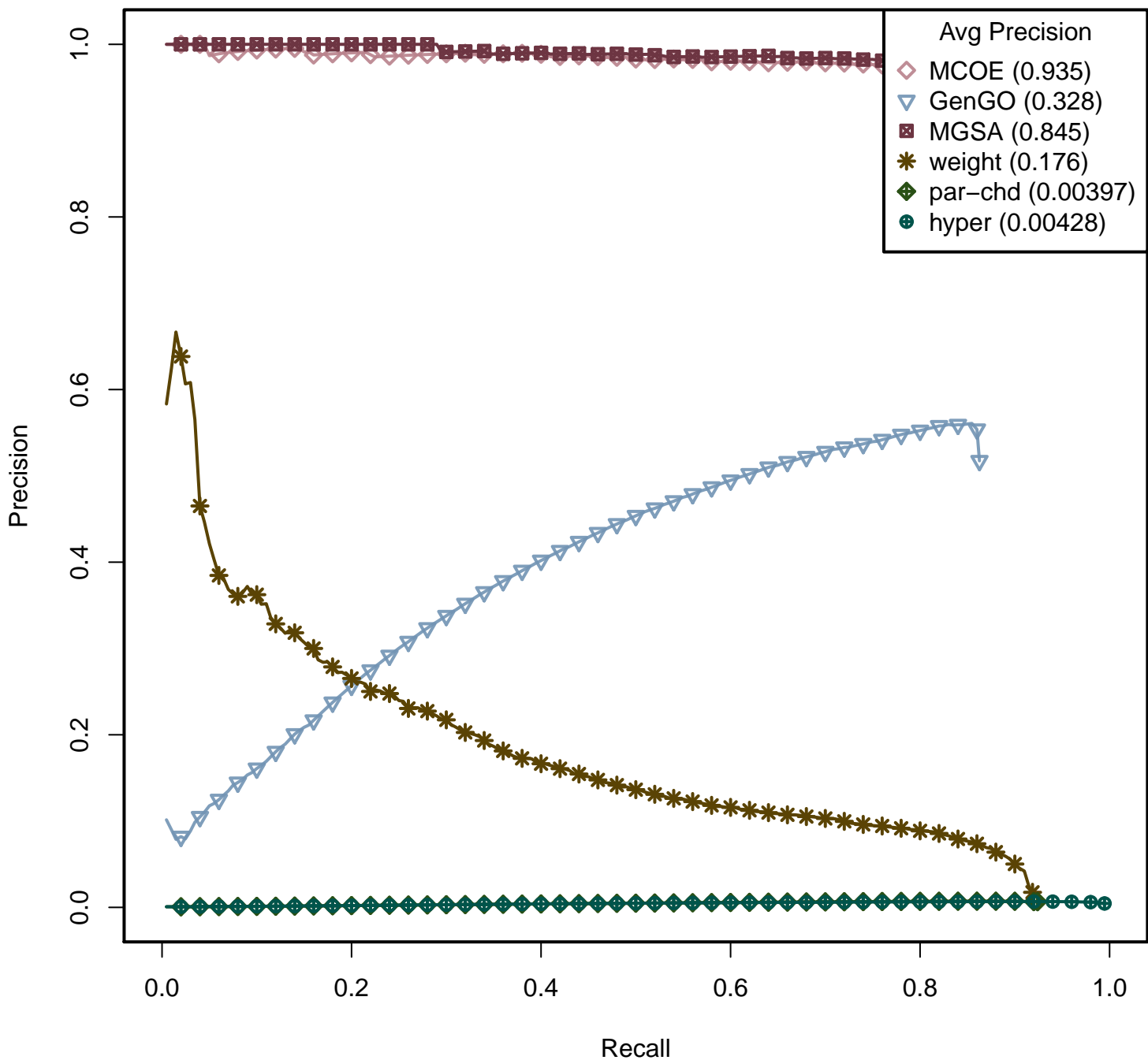

Supplement: Additional File 4 — Benchmarking results on simulated Homo sapiens data sets for false positive rate (q) of 0.01 and false negative rate (1-p) of 0.1. [file 1471-2105-13-23-S4.PDF]

A) Precision/Recall:  $q=0.4, (1-p)=0.25, \sigma=\text{false}$

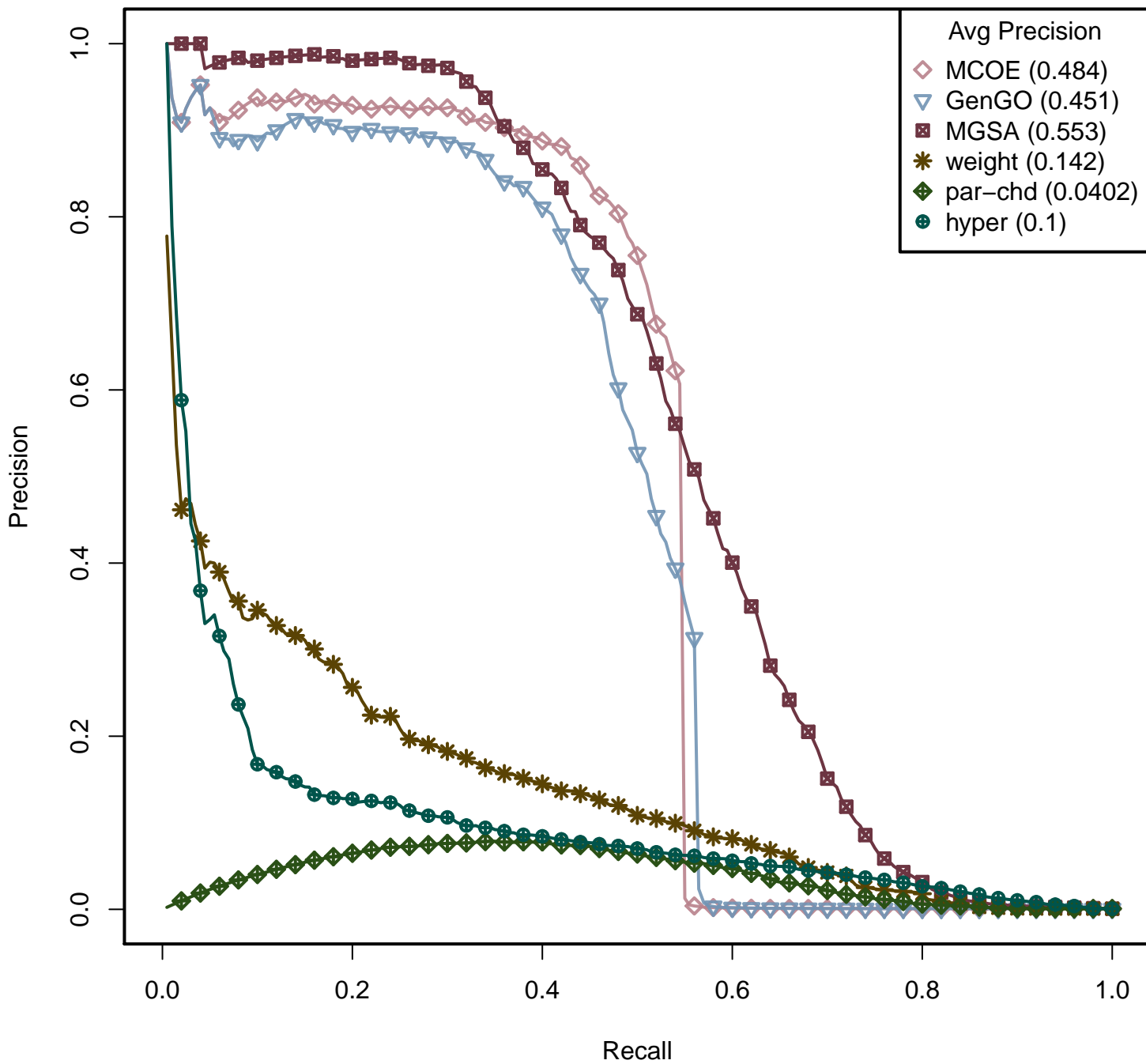

B) Precision/Recall:  $q=0.4, (1-p)=0.25, \sigma=\text{true}$

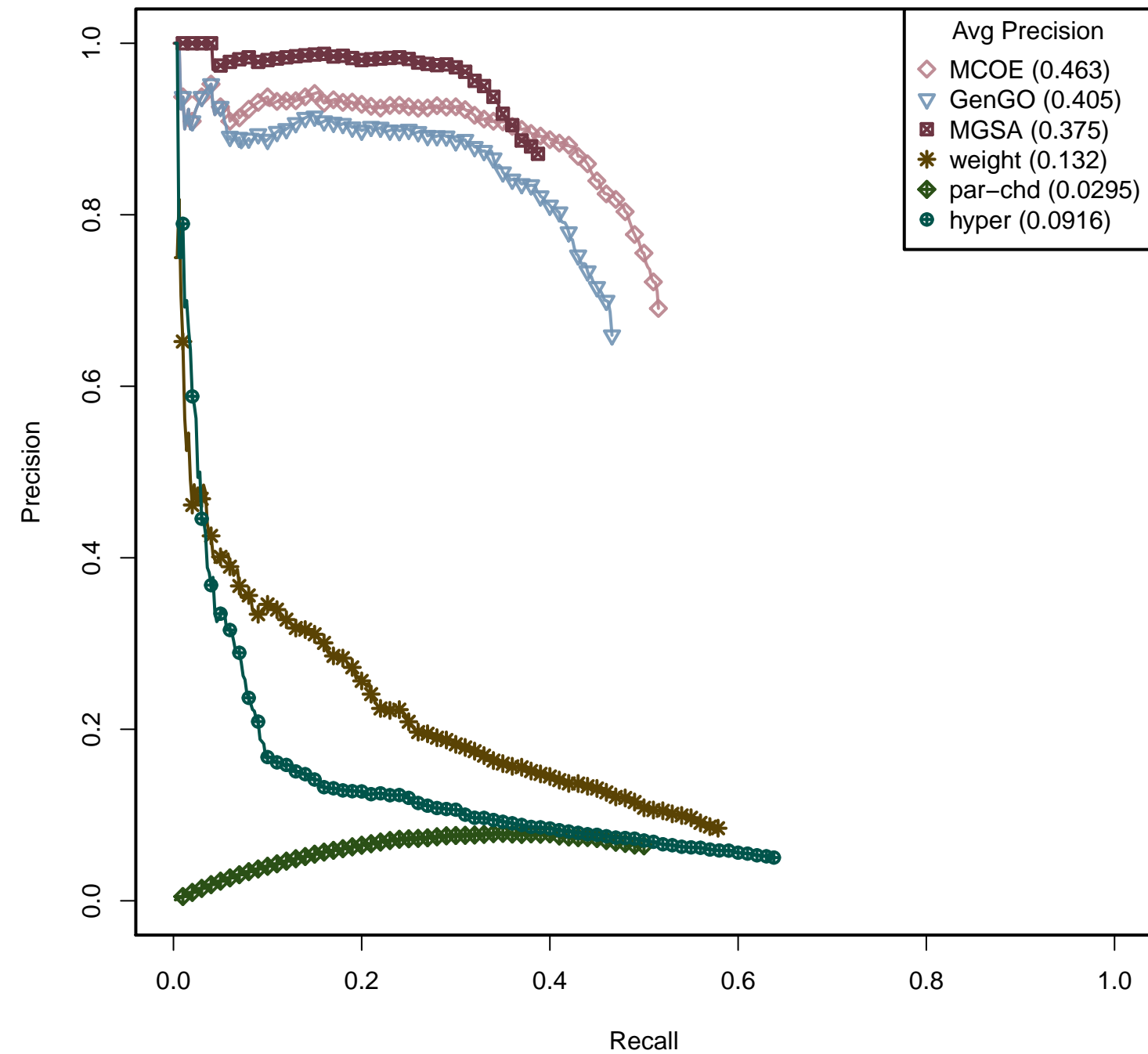

Supplement: Additional File 5 — Benchmarking results on simulated Escherichia coli data sets for false positive rate (q) of 0.4 and false negative rate (1-p) of 0.25. [file 1471-2105-13-23-S5.PDF]

A) Precision/Recall:  $q=0.4, (1-p)=0.25, \sigma=\text{false}$

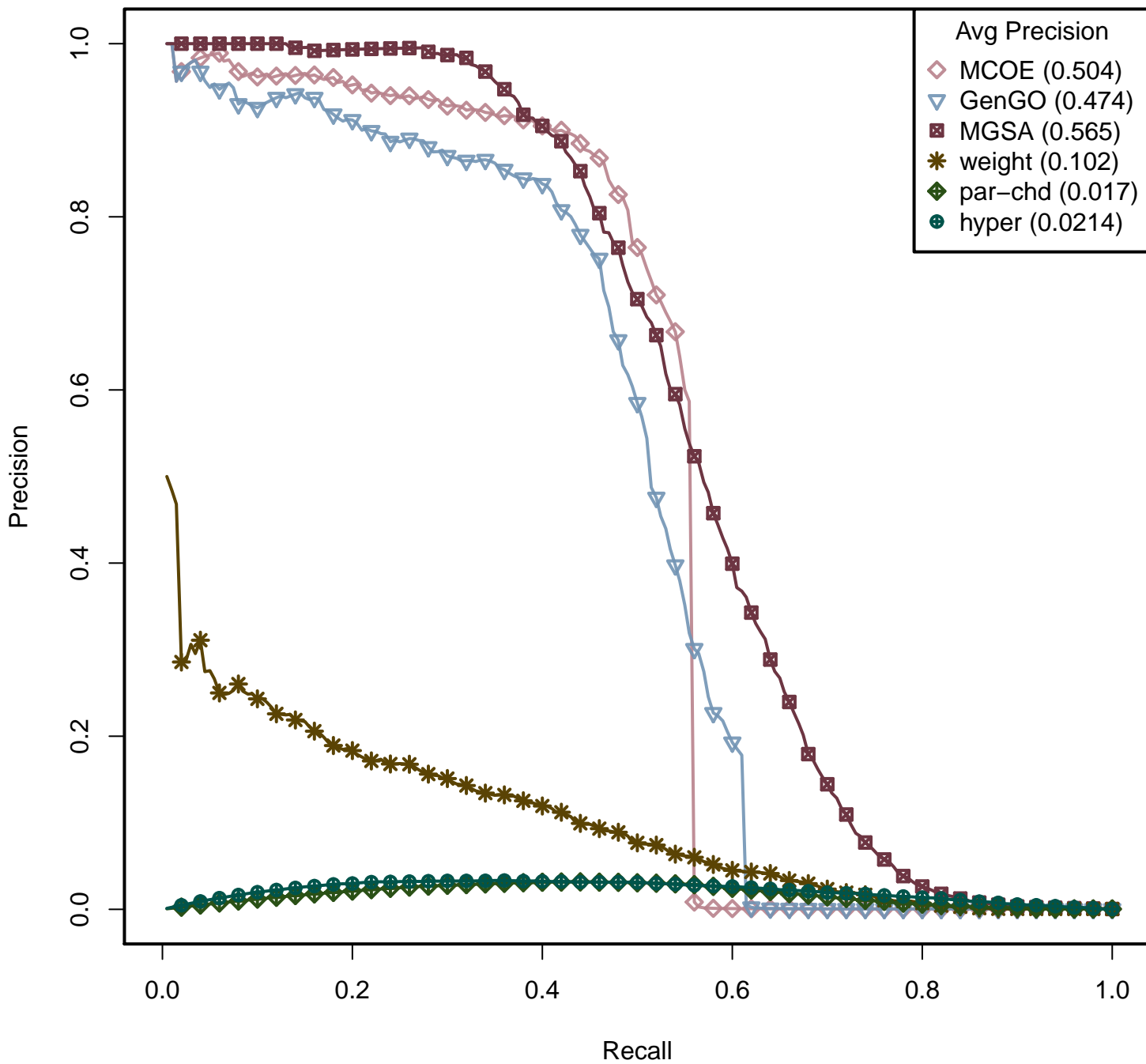

B) Precision/Recall:  $q=0.4, (1-p)=0.25, \sigma=\text{true}$

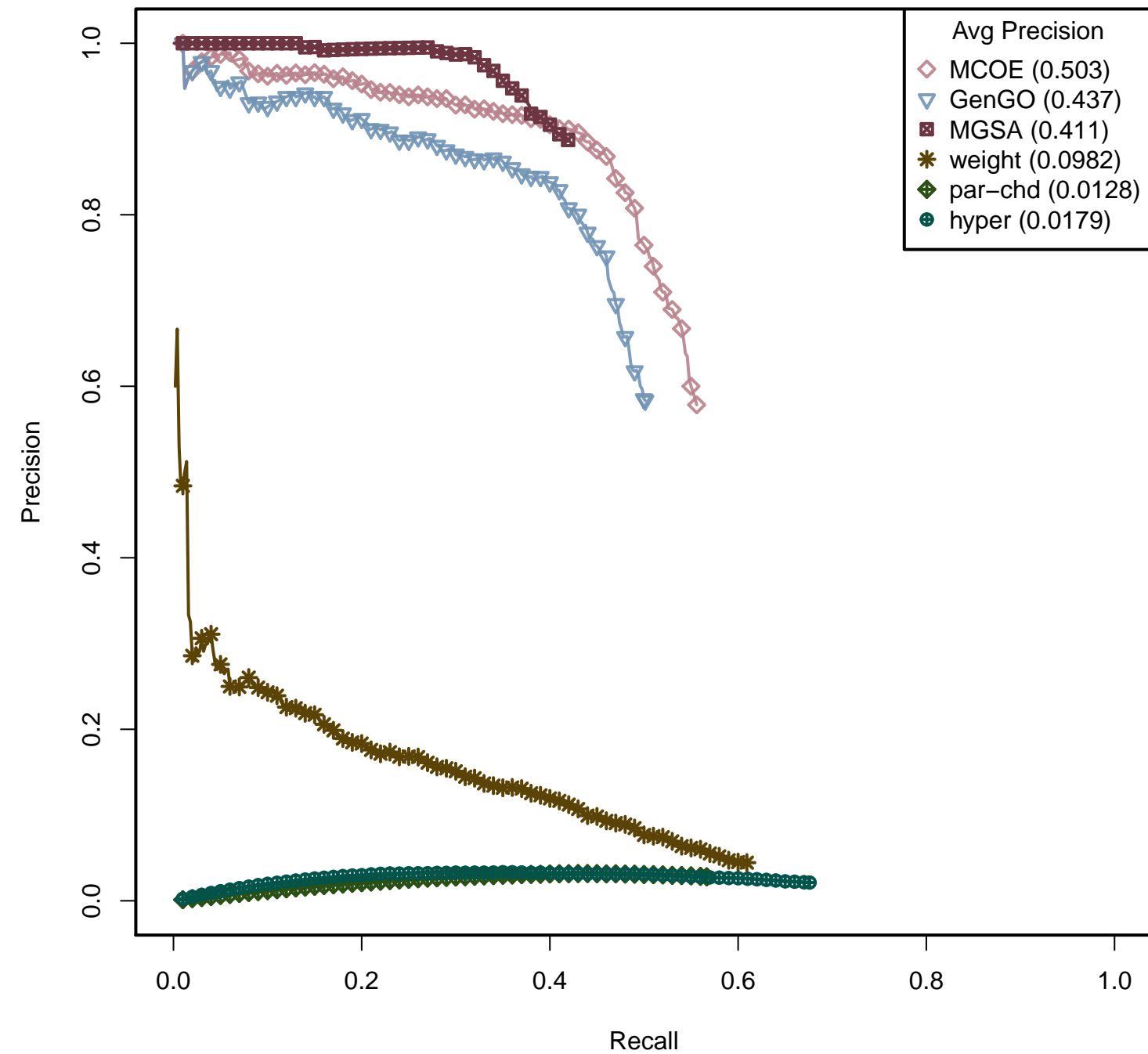

Supplement: Additional File 6 — Benchmarking results on simulated Drosophila Melanogaster data sets for false positive rate (q) of 0.4 and false negative rate (1-p) of 0.25. [file 1471-2105-13-23-S6.PDF]

A) Precision/Recall:  $q=0.4, (1-p)=0.25, \sigma=\text{false}$

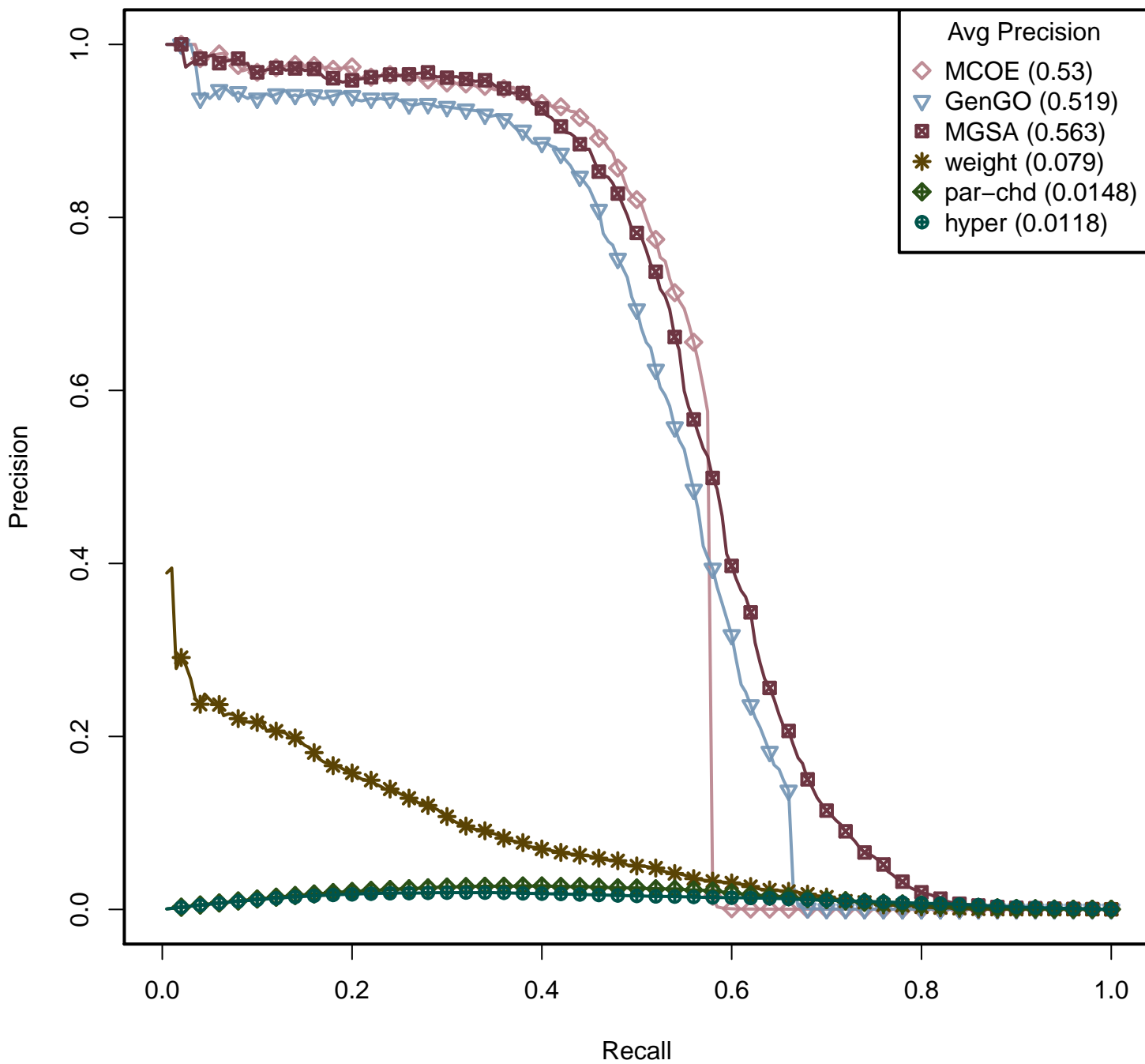

B) Precision/Recall:  $q=0.4, (1-p)=0.25, \sigma=\text{true}$

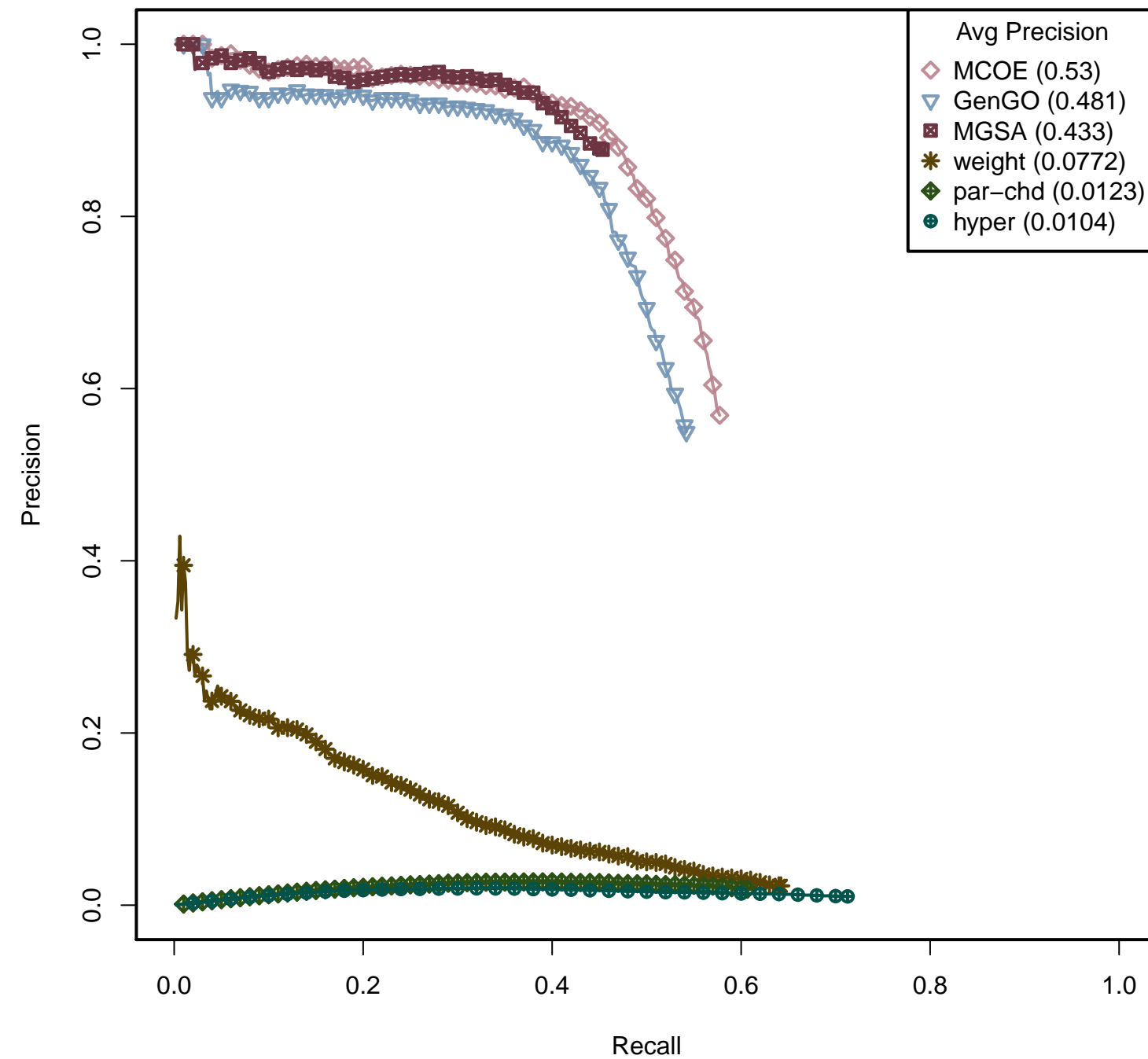

Supplement: Additional File 7 — Benchmarking results on simulated Homo sapiens data sets for false positive rate (q) of 0.4 and false negative rate (1-p) of 0.25. [file 1471-2105-13-23-S7.PDF]

A) Precision/Recall:  $q=0.1, (1-p)=0.1, \sigma=\text{false}$

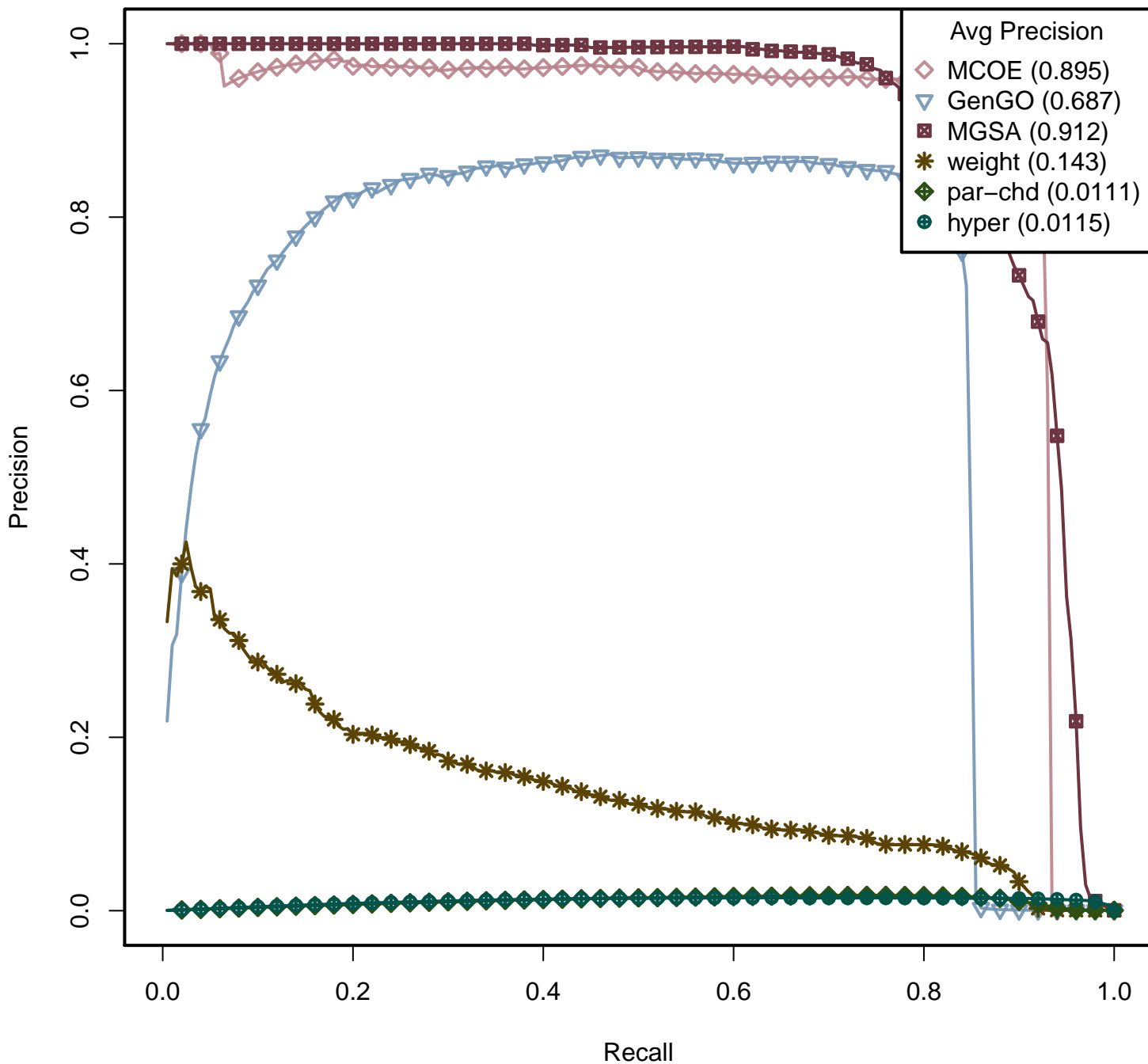

B) Precision/Recall:  $q=0.1, (1-p)=0.1, \sigma=\text{true}$

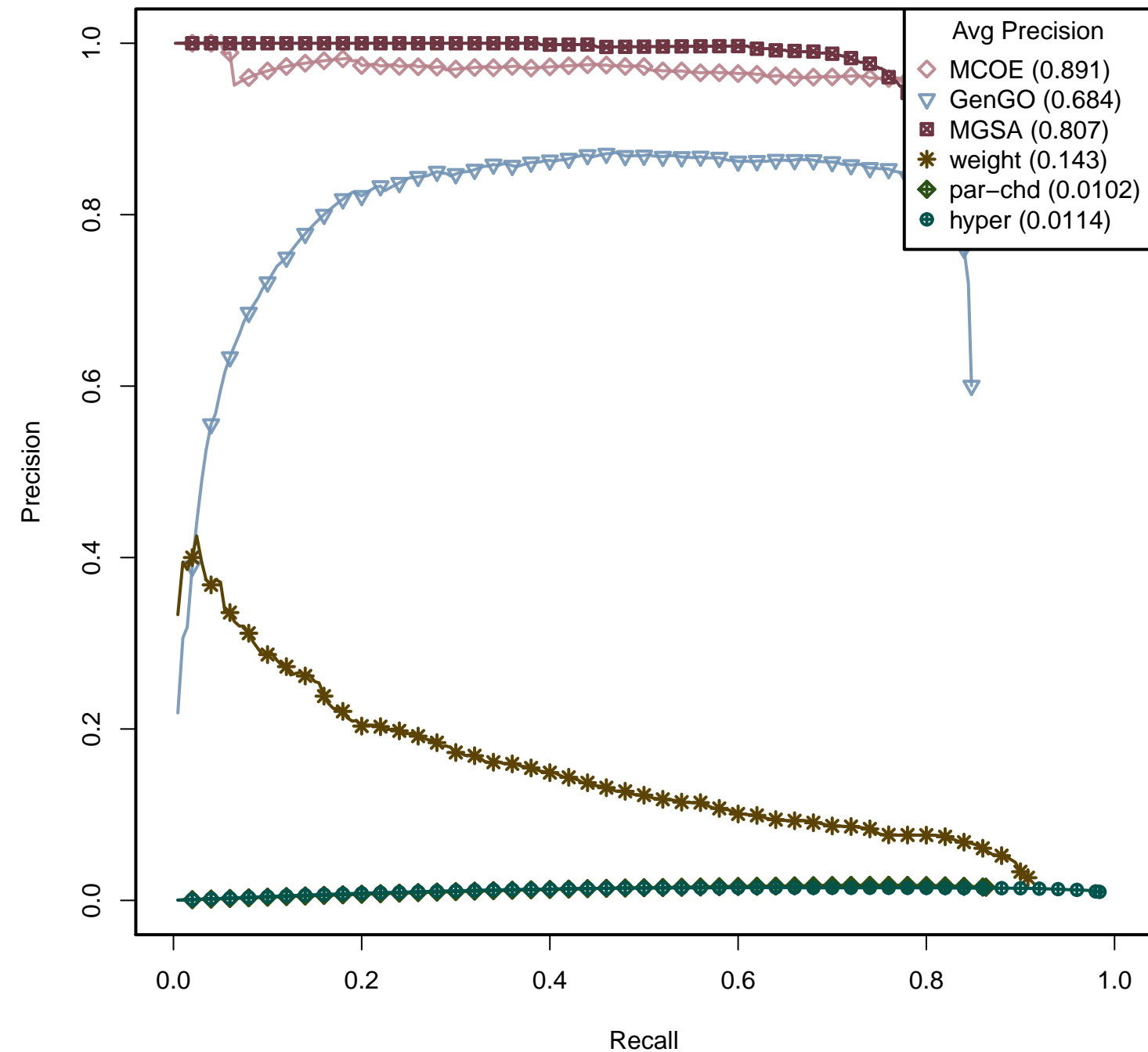

Supplement: Additional File 8 — Benchmarking results on simulated Escherichia coli data sets for false positive rate (q) of 0.1 and false negative rate (1-p) of 0.1. [file 1471-2105-13-23-S8.PDF]

A) Precision/Recall:  $q=0.1, (1-p)=0.1, \sigma=false$

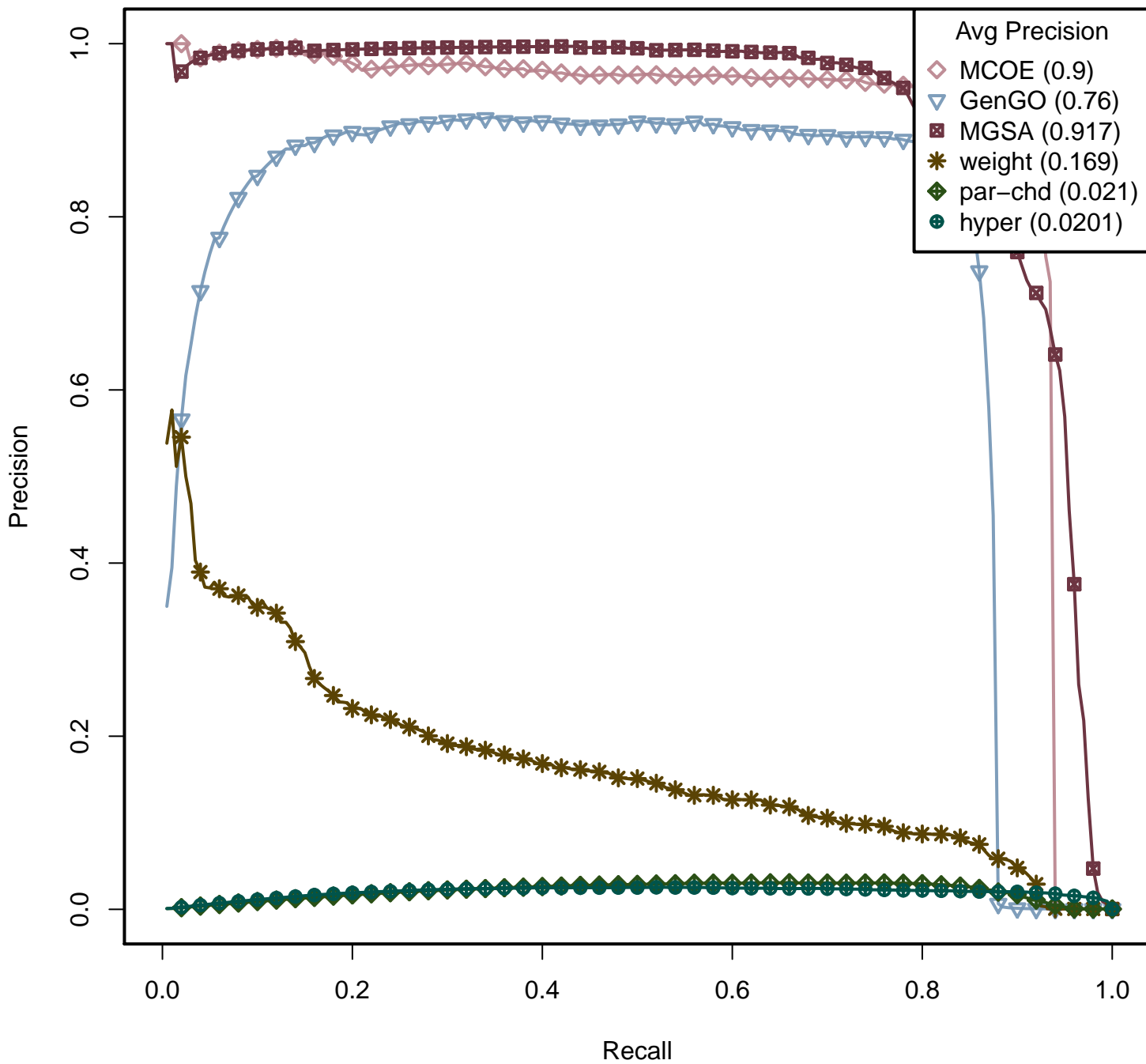

B) Precision/Recall:  $q=0.1, (1-p)=0.1, \sigma=true$

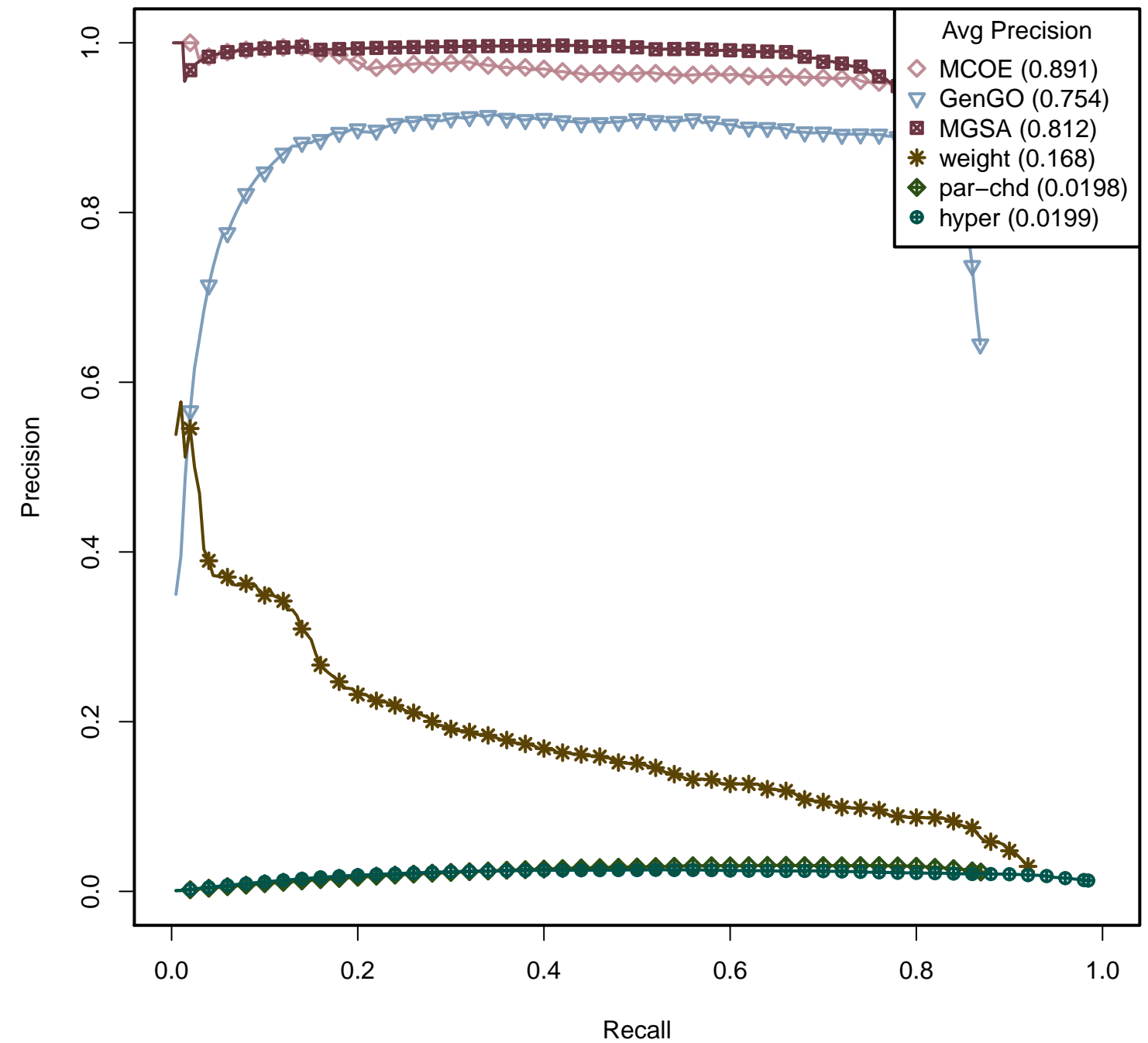

Supplement: Additional File 9 — Benchmarking results on simulated Drosophila Melanogaster data sets for false positive rate (q) of 0.1 and false negative rate (1-p) of 0.1. [file 1471-2105-13-23-S9.PDF]

A) Precision/Recall:  $q=0.1, (1-p)=0.1, \sigma=false$

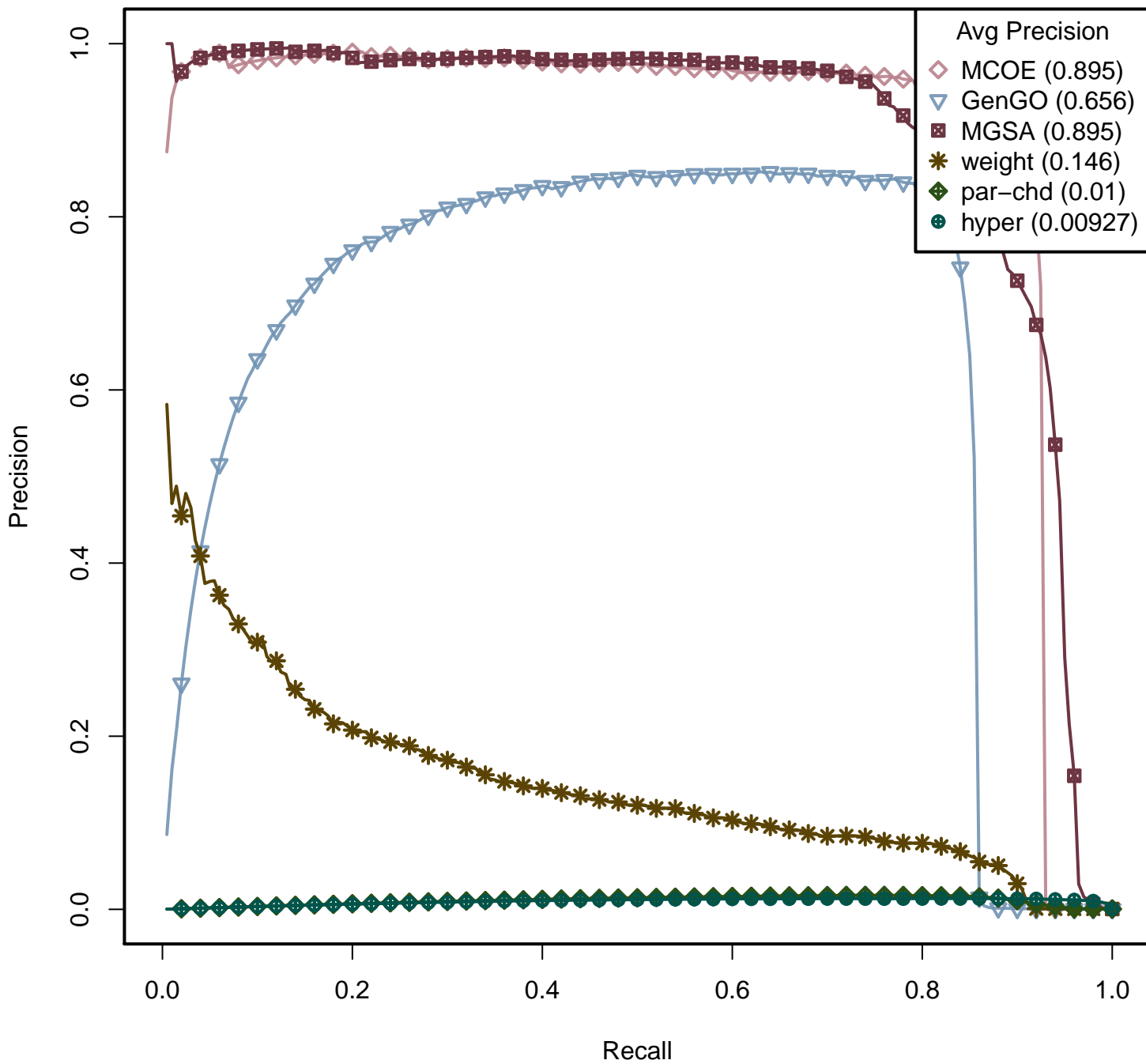

B) Precision/Recall:  $q=0.1, (1-p)=0.1, \sigma=true$

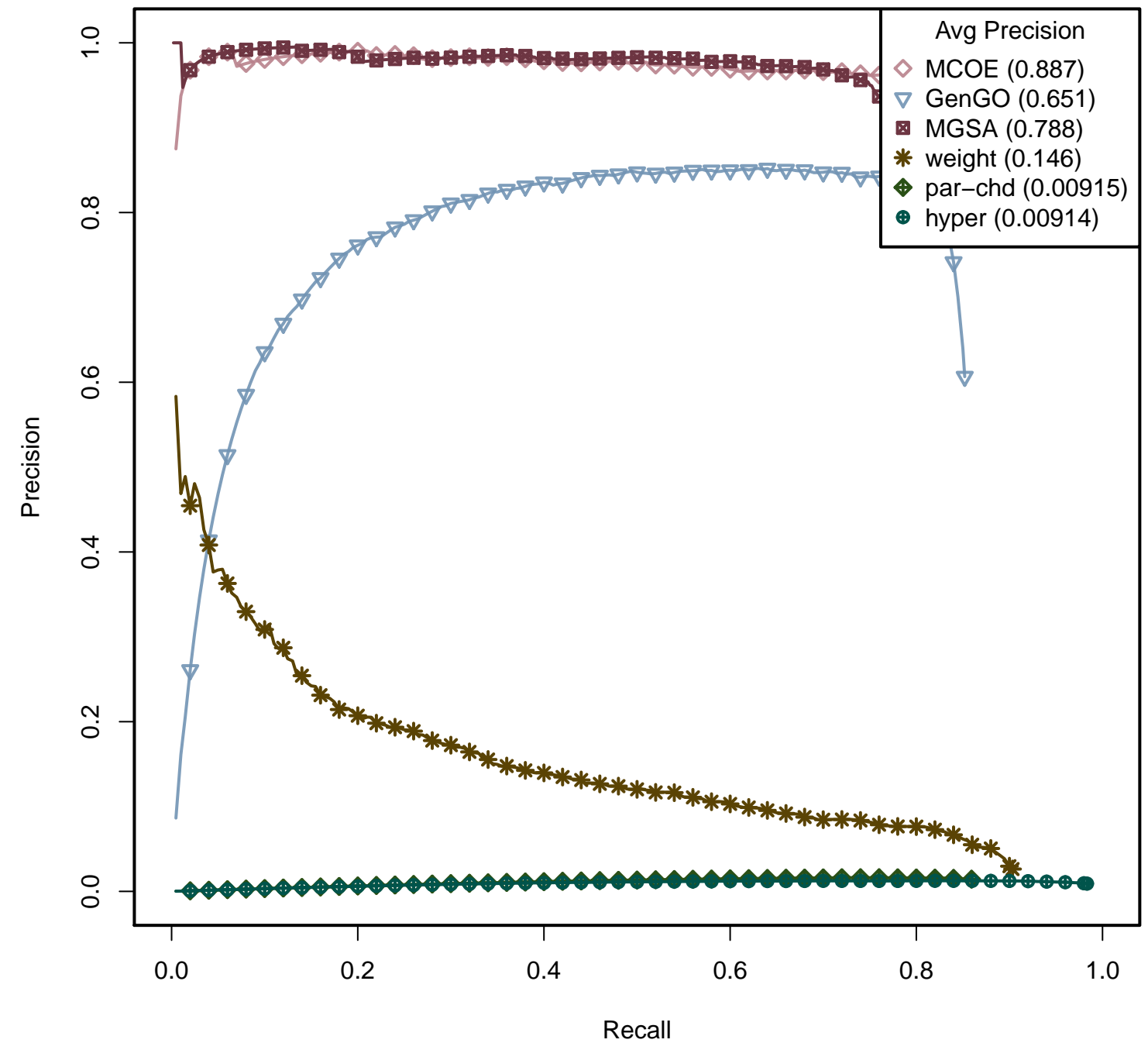

Supplement: Additional File 10 — Benchmarking results on simulated Homo sapiens data sets for false positive rate (q) of 0.1 and false negative rate (1-p) of 0.1. [file 1471-2105-13-23-S10.PDF]

A) Precision/Recall:  $q=0.1, (1-p)=0.4, \sigma=\text{false}$

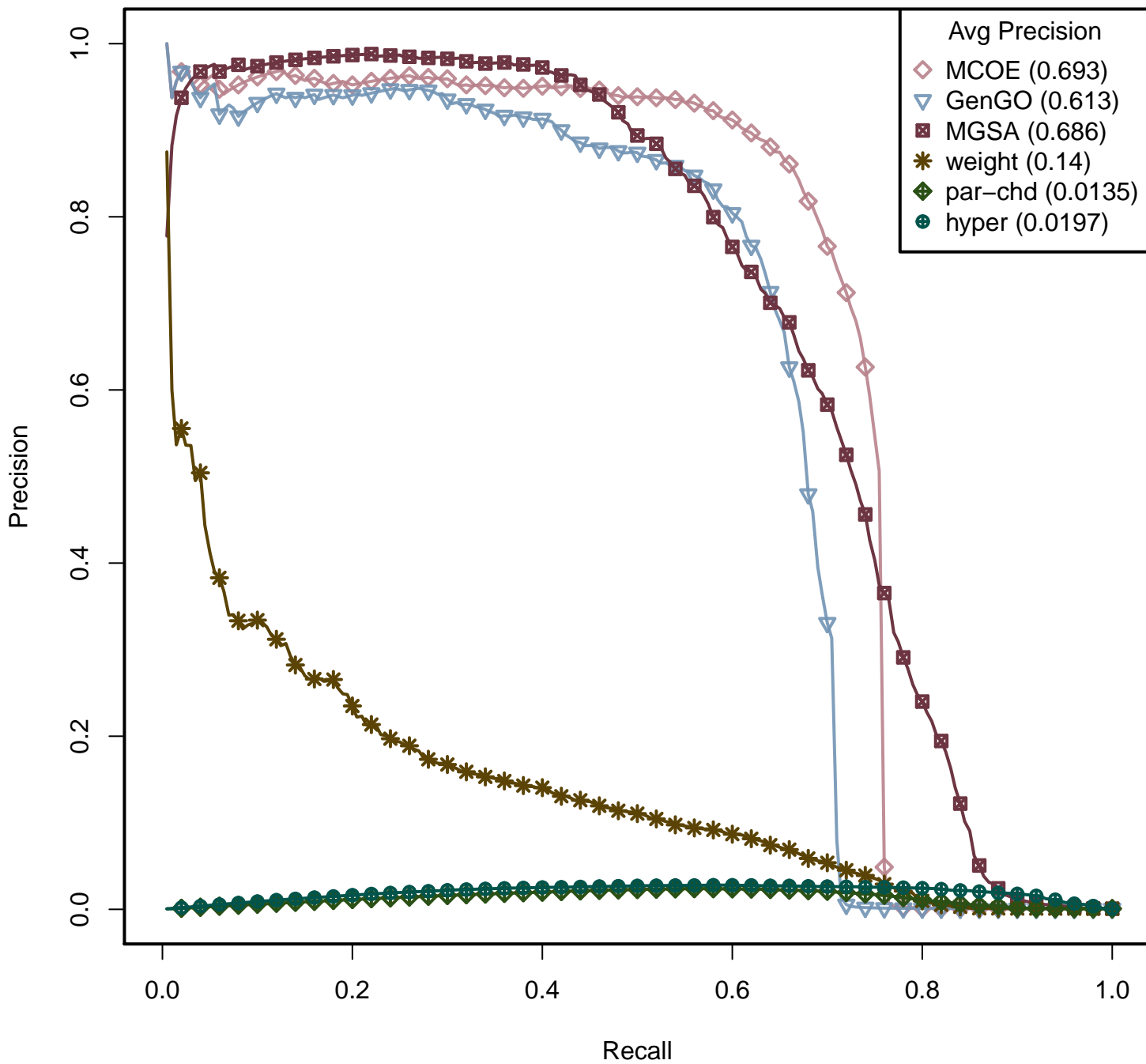

B) Precision/Recall:  $q=0.1, (1-p)=0.4, \sigma=\text{true}$

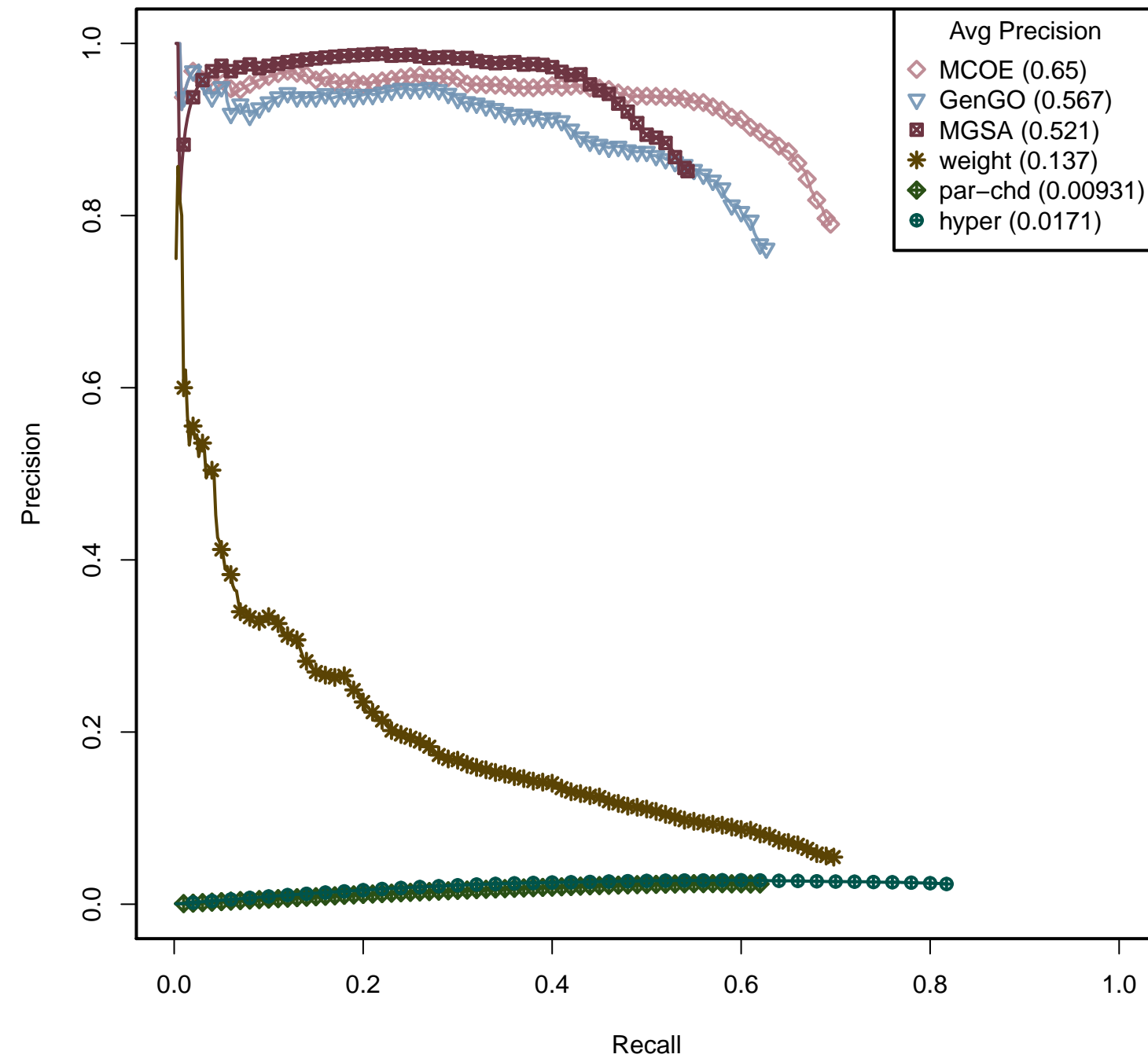

Supplement: Additional File 11 — Benchmarking results on simulated Escherichia coli data sets for false positive rate (q) of 0.1 and false negative rate (1-p) of 0.4. [file 1471-2105-13-23-S11.PDF]

A) Precision/Recall:  $q=0.1, (1-p)=0.4, \sigma=\text{false}$

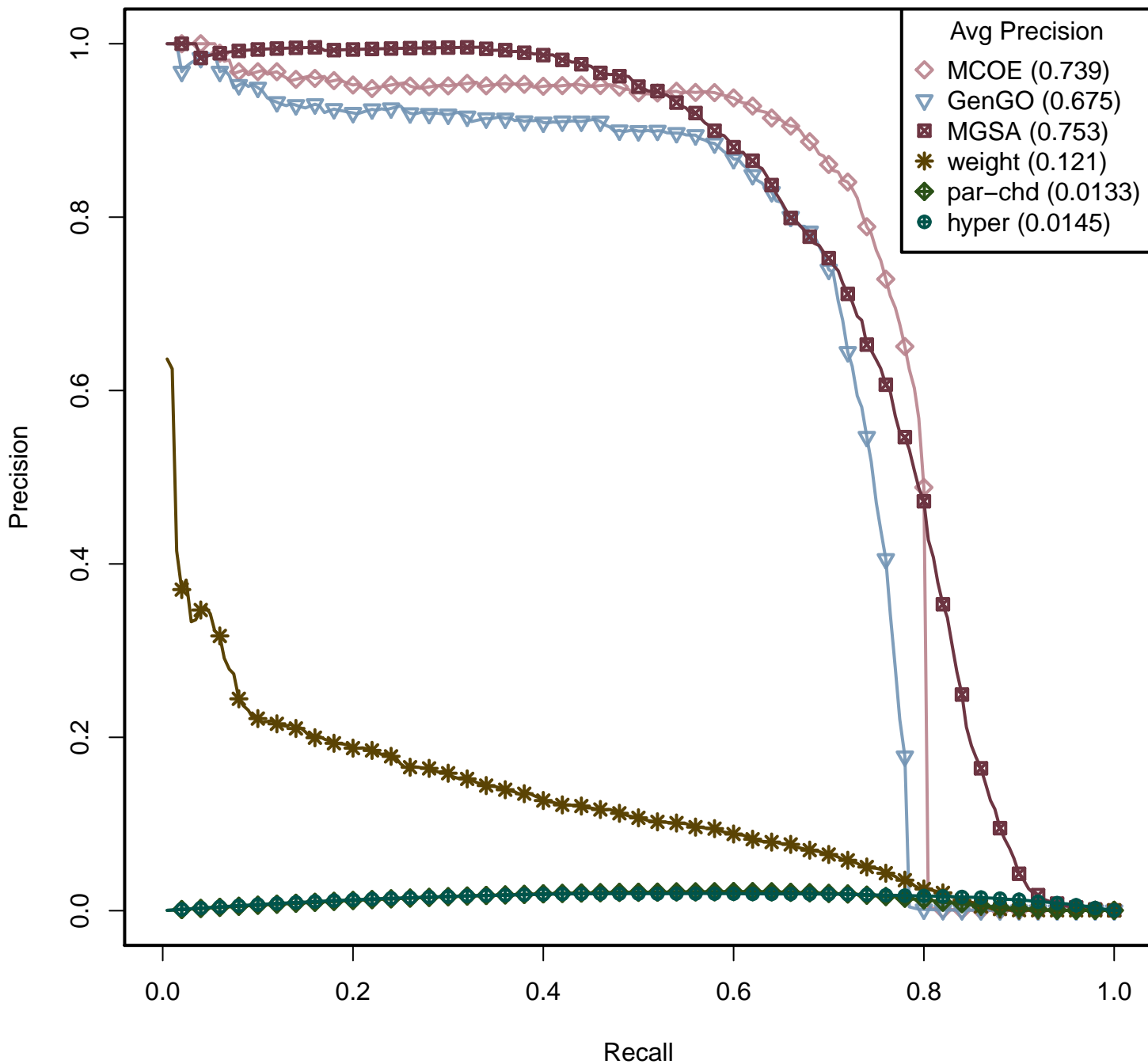

B) Precision/Recall:  $q=0.1, (1-p)=0.4, \sigma=\text{true}$

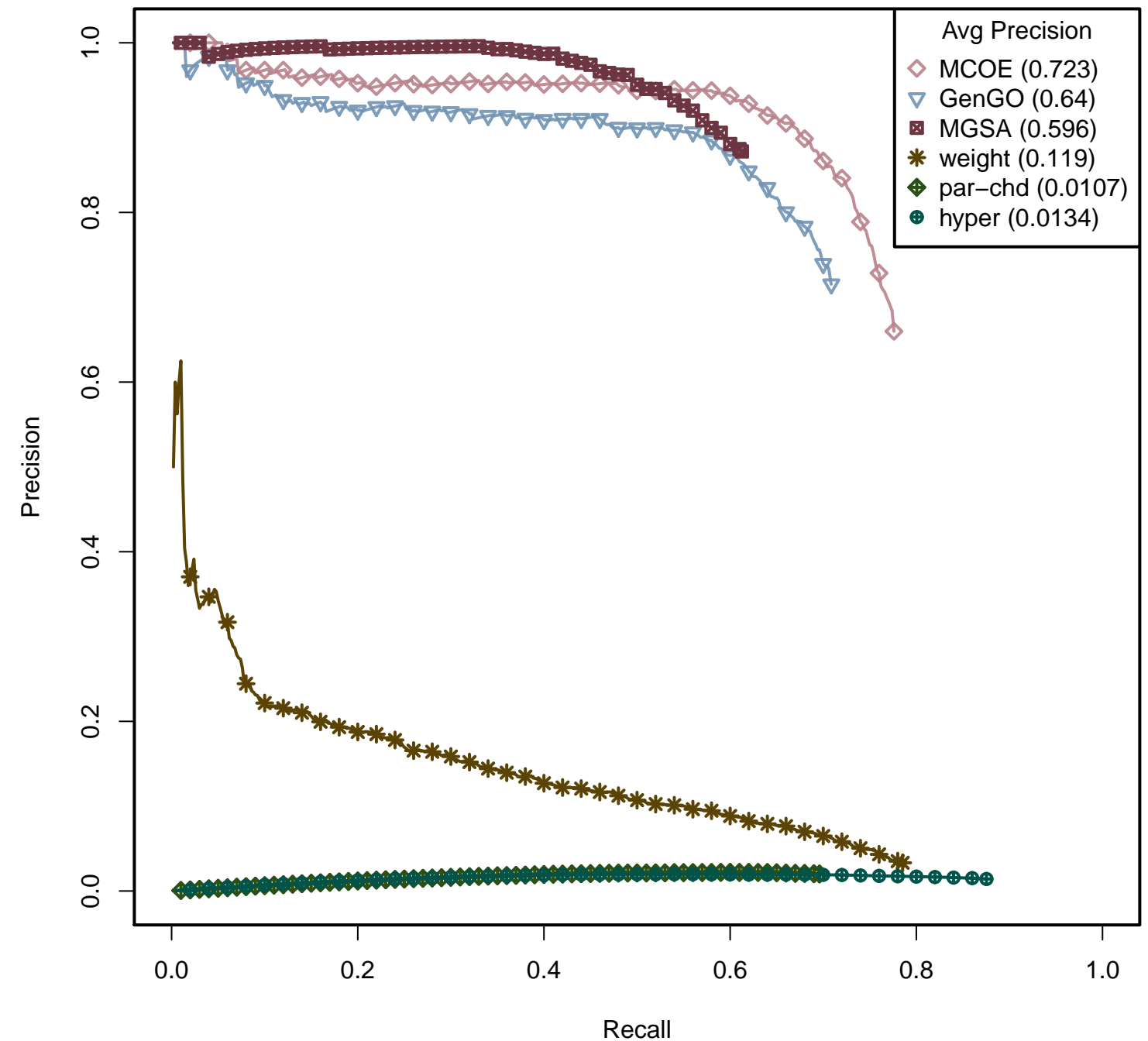

Supplement: Additional File 12 — Benchmarking results on simulated Drosophila Melanogaster data sets for false positive rate (q) of 0.1 and false negative rate (1-p) of 0.4. [file 1471-2105-13-23-S12.PDF]

A) Precision/Recall:  $q=0.1, (1-p)=0.4, \sigma=\text{false}$

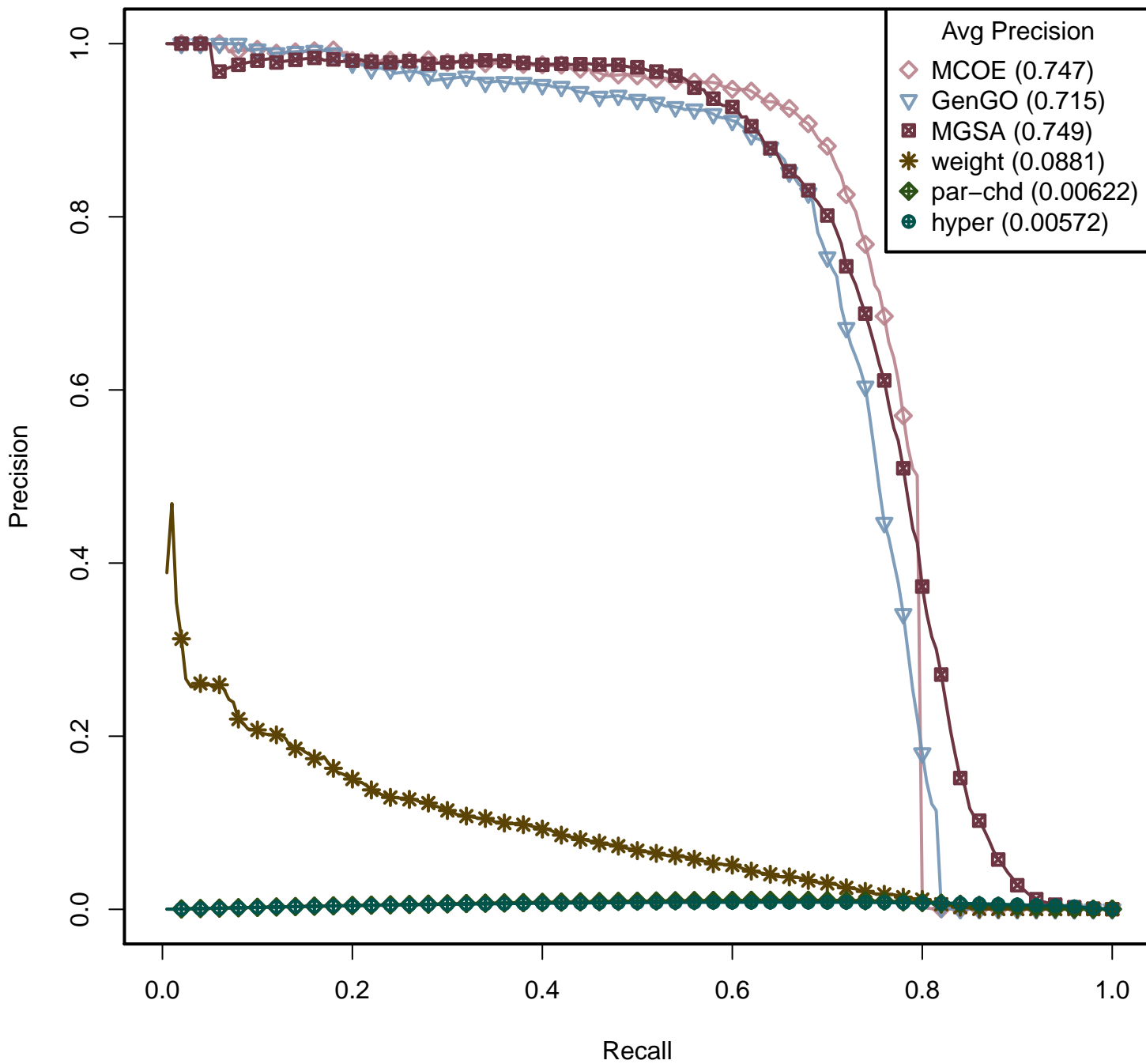

B) Precision/Recall:  $q=0.1, (1-p)=0.4, \sigma=\text{true}$

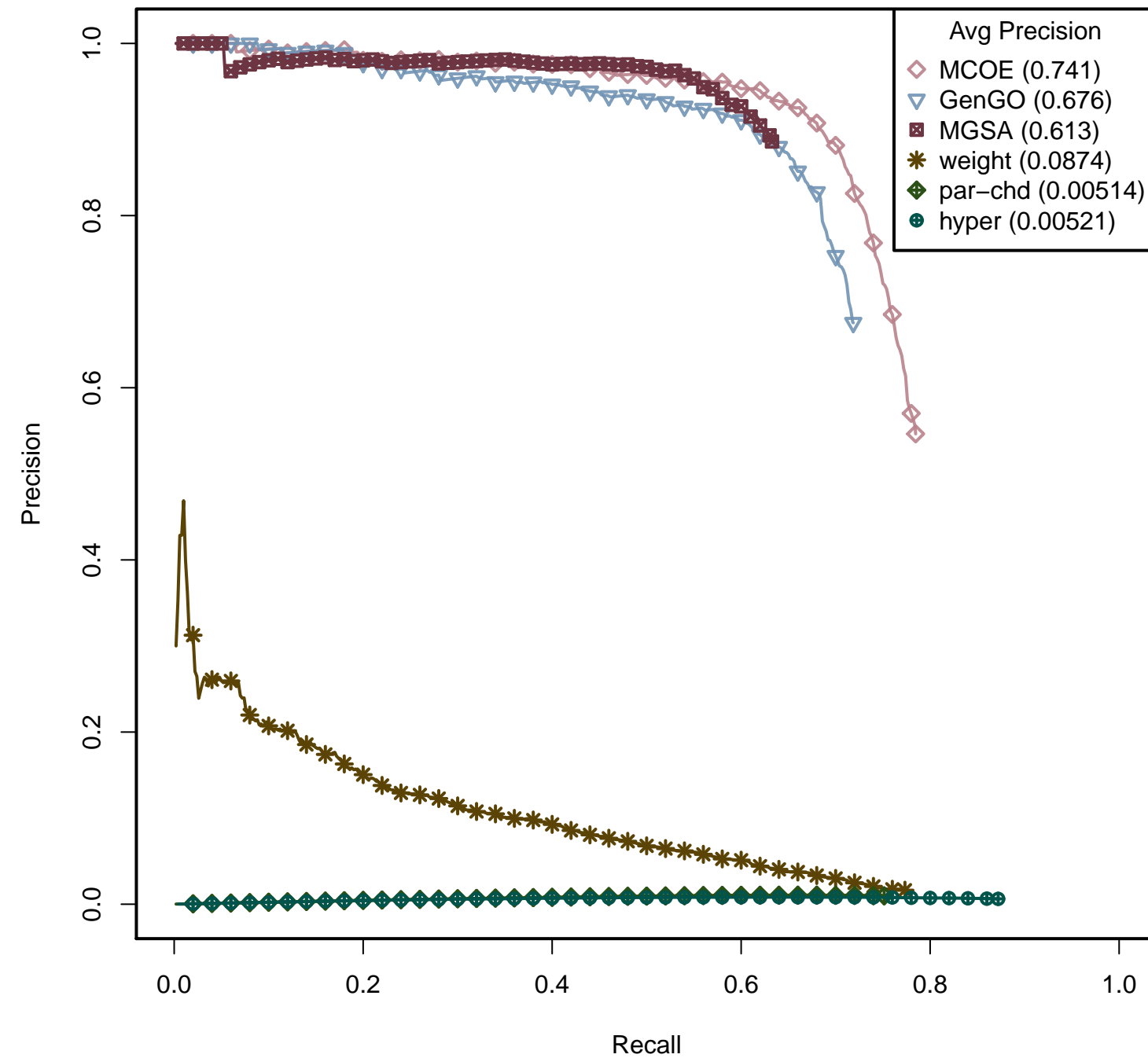

Supplement: Additional File 13 — Benchmarking results on simulated Homo sapiens data sets for false positive rate (q) of 0.1 and false negative rate (1-p) of 0.4. [file 1471-2105-13-23-S13.PDF]
