# Supplementary material for: Markov Chain Ontology Analysis (MCOA)
Source: BMC Bioinformatics. 2012 Feb 3;13:23. doi: 10.1186/1471-2105-13-23 (PMC3329418; doi:10.1186/1471-2105-13-23)
Supplement: Additional File 14 — Relative execution time statistics on simulated Homo sapiens data. [file 1471-2105-13-23-S14.PDF]

Markov Chain Ontology Analysis - Supplementary Material: Execution time of enrichment methods on simulated data

Relative execution time statistics for all evaluated methods during benchmark evaluation on simulated Homo sapiens data (see paper for details on creation of simulated GO datasets).

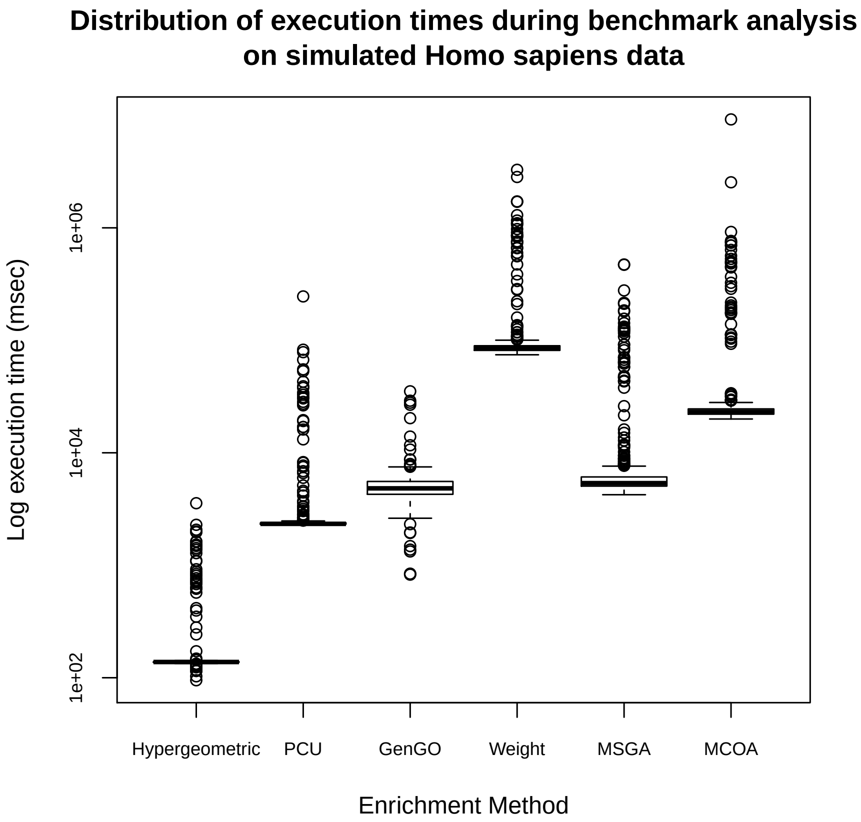

Descriptive statistics for each enrichment method (times in msec):

	Hypergeometric	PCU	GenGO	Weight	MSGA	MCOA
Min.	95.00	2273.00	826.00	74390.00	4238.00	19970.00
1st Qu.	137.00	2318.00	4276.00	81390.00	5056.00	22060.00
Median	138.00	2335.00	4828.00	84330.00	5374.00	23100.00
Mean	202.70	4384.00	5137.00	140000.00	13960.00	69810.00
3rd Qu.	139.00	2385.00	5560.00	89470.00	6090.00	24550.00
Max.	3564.00	245700.00	35220.00	3283000.00	472500.00	9211000.00
