# Supplementary material for: Markov Chain Ontology Analysis (MCOA)
Source: BMC Bioinformatics. 2012 Feb 3;13:23. doi: 10.1186/1471-2105-13-23 (PMC3329418; doi:10.1186/1471-2105-13-23)
Supplement: Additional File 19 — Research linking top ten GO terms returned by MCOA on GEO dataset GDS3129 and Parkinson's disease. [file 1471-2105-13-23-S19.PDF]

Markov Chain Ontology Analysis (MCOA) - Supplementary Material

Analysis of Gene Expression Omnibus (GEO) Dataset GDS3129

All of the top results returned by MCOA, as shown in Table 1 of the main manuscript, are specific, non-overlapping and associated with recently published findings linking the associated biological process, molecular function or cellular component to Parkinson's disease:

1. ***regulation of osteoclast differentiation****:* Is supported by research linking Parkinson's with low bone density/osteoporosis[1, 2] as well as the finding of rheumatoid as a comorbidity[3].
2. ***glucose homeostasis***: Is supported by the link between Parkinson's and cortical hypometabolism[4, 5] as well as the association between insulin gylcation, glucose homeostasis and Parkinson's [6].
3. ***lymphocyte mediated immunity***: Is supported by research that links neurodegeneration in a mouse model of Parkinson's with the presence of CD4+ lymphocytes in the brain[7].
4. ***regulation of angiogenesis***: Is supported by research linking Parkinson's disease with angiogensis[8].
5. ***cilium axoneme***: Is supported by research that ties Parkinson's disease with defects in microtubule cytokeleton[9, 10].
6. ***negative regulation of transmembrane transport***: Is supported by findings that Parkinson's disease is associated with aging and deterioriation of the nuclear pore complex, leaking of cytoplasmic proteins into the nucleus and the build-up of tublin filaments inside the nucleus[11].

7. ***steroid hormone receptor signaling pathway:*** Is supported by research linking oestrogen receptors and Parkinson’s disease[12].
8. ***ras guanyl-nucleotide exchange factor activity***: Is supported by research that associates movement disorders in Parkinson’s patients with upregulation of RasGRP (a Ras guanyl-nucleotide exchange factor)[13].
9. ***collagen***: Is supported by the finding that Parkinson’s patients frequently suffer from hypophonia, which is treated with collagen[14]. A type of collagen that is attacked by a skin auto-immune disease associated with neurological disorders has also been found inside neurons[15].
10. ***cytokine receptor activity***: Is supported by research that discovered increased levels of proinflammatory cytokines are found in the brains of Parkinson’s patients[16].

References

1. Invernizzi M, Carda S, Viscontini GS, Cisari C: **Osteoporosis in Parkinson’s disease**. *Parkinsonism Relat. Disord.* 2009, **15**:339-346.

2. Gnädinger M, Mellinghoff H-U, Kaelin-Lang A: **Parkinson’s disease and the bones**. *Swiss Med Wkly* 2011, **141**:w13154.

3. Gupta M, Cheung C-L, Hsu Y-H, Demissie S, Cupples LA, Kiel DP, Karasik D: **Identification of homogeneous genetic architecture of multiple genetically correlated traits by block clustering of genome-wide associations**. *J. Bone Miner. Res.* 2011, **26**:1261-1271.

4. Borghammer P, Chakravarty M, Jonsdottir KY, Sato N, Matsuda H, Ito K, Arahata Y, Kato T, Gjedde A: **Cortical hypometabolism and hypoperfusion in Parkinson’s disease is extensive: probably even at early disease stages**. *Brain Struct Funct* 2010, **214**:303-317.

5. Pappatà S, Santangelo G, Aarsland D, Vicidomini C, Longo K, Bronnick K, Amboni M, Erro R, Vitale C, Caprio MG, Pellecchia MT, Brunetti A, De Michele G, Salvatore M, Barone P: **Mild cognitive impairment in drug-naive patients with PD is associated with cerebral hypometabolism**. *Neurology* 2011, **77**:1357-1362.

6. Oliveira LMA, Lages A, Gomes RA, Neves H, Família C, Coelho AV, Quintas A: **Insulin glycation by methylglyoxal results in native-like aggregation and inhibition of fibril formation**. *BMC Biochem.* 2011, **12**:41.

7. Brochard V, Combadière B, Prigent A, Laouar Y, Perrin A, Beray-Berthat V, Bonduelle O, Alvarez-Fischer D, Callebert J, Launay J-M, Duyckaerts C, Flavell RA, Hirsch EC, Hunot S: **Infiltration of CD4+ lymphocytes into the brain contributes to neurodegeneration in a mouse model of Parkinson disease**. *J Clin Invest* 2009, **119**:182-192.

8. Desai Bradaric B, Patel A, Schneider JA, Carvey PM, Hendey B: **Evidence for angiogenesis in Parkinson's disease, incidental Lewy body disease, and progressive supranuclear palsy**. *Journal of Neural Transmission (Vienna, Austria: 1996)* 2011.

9. Janke C, Chloë Bulinski J: **Post-translational regulation of the microtubule cytoskeleton: mechanisms and functions**. *Nat. Rev. Mol. Cell Biol.* 2011, **12**:773-786.

10. Rogowski K, van Dijk J, Magiera MM, Bosc C, Deloulme J-C, Bosson A, Peris L, Gold ND, Lacroix B, Grau MB, Bec N, Larroque C, Desagher S, Holzer M, Andrieux A, Moutin M-J, Janke C: **A family of protein-deglutamylating enzymes associated with neurodegeneration**. *Cell* 2010, **143**:564-578.

11. Hetzer MW: **The role of the nuclear pore complex in aging of post-mitotic cells**. *Aging (Albany NY)* 2010, **2**:74-75.

12. Al Sweidi S, Sánchez MG, Bourque M, Morissette M, Dluzen D, Di Paolo T: **Oestrogen receptors and signalling pathways: implications for neuroprotective effects of sex steroids in parkinson's disease**. *Journal of Neuroendocrinology* 2011.

13. Crittenden JR, Cantuti-Castelvetri I, Saka E, Keller-McGandy CE, Hernandez LF, Kett LR, Young AB, Standaert DG, Graybiel AM: **Dysregulation of CalDAG-GEFI and CalDAG-GEFII predicts the severity of motor side-effects induced by anti-parkinsonian therapy**. *Proc. Natl. Acad. Sci. U.S.A.* 2009, **106**:2892-2896.

14. Berke GS, Gerratt B, Kreiman J, Jackson K: **Treatment of Parkinson Hypophonia With Percutaneous Collagen Augmentation**. *The Laryngoscope* 1999, **109**:1295-1299.

15. Seppänen A, Autio-Harmainen H, Alafuzoff I, Särkioja T, Veijola J, Hurskainen T, Bruckner-Tuderman L, Tasanen K, Majamaa K: **Collagen XVII is expressed in human CNS neurons**. *Matrix Biology* 2006, **25**:185-188.

16. Reale M, Iarlori C, Thomas A, Gambi D, Perfetti B, Di Nicola M, Onofrj M: **Peripheral cytokines profile in Parkinson's disease**. *Brain Behav. Immun.* 2009, **23**:55-63.
